# The RNA pseudoknots in foot-and-mouth disease virus are dispensable for genome replication, but essential for the production of infectious virus

**DOI:** 10.1371/journal.ppat.1010589

**Published:** 2022-06-06

**Authors:** Joseph C. Ward, Lidia Lasecka-Dykes, Chris Neil, Oluwapelumi O. Adeyemi, Sarah Gold, Niall McLean-Pell, Caroline Wright, Morgan R. Herod, David Kealy, Emma Warner, Terry Jackson, Donald P. King, Tobias J. Tuthill, David J. Rowlands, Nicola J. Stonehouse

**Affiliations:** 1 School of Molecular and Cellular Biology, Faculty of Biological Sciences and Astbury Centre for Structural Molecular Biology, University of Leeds, Leeds, United Kingdom; 2 Pirbright Institute, Ash Road, Pirbright, Surrey, United Kingdom; Chang Gung University, TAIWAN

## Abstract

Non-coding regions of viral RNA (vRNA) genomes are critically important in the regulation of gene expression. In particular, pseudoknot (PK) structures, which are present in a wide range of RNA molecules, have a variety of roles. The 5′ untranslated region (5′ UTR) of foot-and-mouth disease virus (FMDV) vRNA is considerably longer than in other viruses from the picornavirus family and consists of a number of distinctive structural motifs that includes multiple (2, 3 or 4 depending on the virus strain) putative PKs linked in tandem. The role(s) of the PKs in the FMDV infection are not fully understood. Here, using bioinformatics, sub-genomic replicons and recombinant viruses we have investigated the structural conservation and importance of the PKs in the FMDV lifecycle. Our results show that despite the conservation of two or more PKs across all FMDVs, a replicon lacking PKs was replication competent, albeit at reduced levels. Furthermore, in competition experiments, GFP FMDV replicons with less than two (0 or 1) PK structures were outcompeted by a mCherry FMDV wt replicon that had 4 PKs, whereas GFP replicons with 2 or 4 PKs were not. This apparent replicative advantage offered by the additional PKs correlates with the maintenance of at least two PKs in the genomes of FMDV field isolates. Despite a replicon lacking any PKs retaining the ability to replicate, viruses completely lacking PK were not viable and at least one PK was essential for recovery of infections virus, suggesting a role for the PKs in virion assembly. Thus, our study points to roles for the PKs in both vRNA replication and virion assembly, thereby improving understanding the molecular biology of FMDV replication and the wider roles of PK in RNA functions.

## Author summary

Foot-and-mouth disease (FMD) is a highly contagious viral disease posing a constant threat to the global livestock industry, as it remains endemic in many parts of the world. The development of novel control measures for FMD could have major economic and social value worldwide, however, this will require a deeper understanding of the molecular mechanisms of FMDV replication. A number of features of the viral RNA genome remain poorly understood, including the role of a series of putative pseudoknot (PK) structures, where the RNA is predicted to fold back on itself. In this study, we confirmed the conservation and structure of these PKs in the FMDV genome and showed that the presence of at least one is essential for production of infectious virus particles. However, the presence of two or more PKs was advantageous in replication competition experiments. These results correlate with the observation that genomes of all known FMDV field isolates include at least two PK structures. We suggest roles for the PKs in both replication and virion assembly.

## Introduction

Foot-and-mouth disease virus (FMDV) is a single stranded positive sense RNA virus of the genus *Aphthovirus* in the family *Picornaviridae*. It occurs as seven, antigenically diverse serotypes; A, O, C, Asia 1, Southern African Territories (SAT) 1, SAT 2 and SAT 3, although the type C viruses appear to no longer exist in the wild [1]. FMDV is the causative agent of foot-and-mouth disease (FMD), a highly contagious disease of cloven-hooved animals affecting most notably cattle, pigs, sheep and goats. In addition, wild species, such as African buffalo (*Syncerus caffer*) are thought to be natural reservoirs of the virus and have largely asymptomatic infection [2–4]. Disease outbreaks have serious economic consequences resulting from trade restrictions, reduced productivity and the slaughter of infected and at-risk animals [5]. The 2001 outbreak in the UK caused economic losses equivalent to £15 billion (adjusted for inflation) to the agricultural and tourism sectors. Inactivated virus vaccines are used in countries in which FMD is endemic, but these are often strain-specific and provide little cross protection between serotypes [6]. Antigenic variation, together with the relatively short duration of immunity following vaccination, combine to complicate control of the disease [7]. In addition, the carrier state, in which asymptomatically infected animals continue to harbour virus, may contribute to the spread of FMDV [8–10]. An improved understanding of the viral life cycle may pave the way for the future development of improved vaccines and other novel control measures.

**Fig 1:**
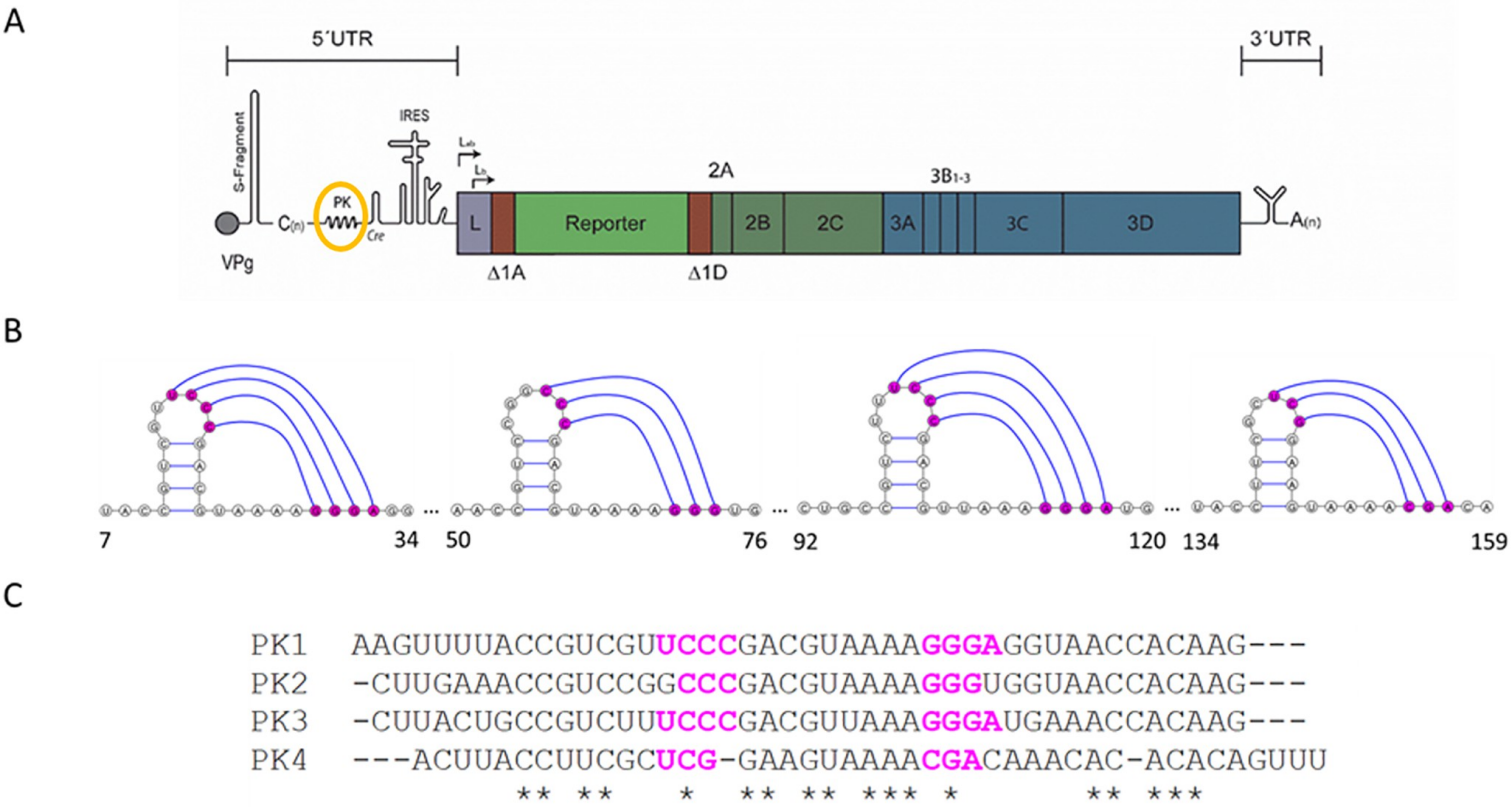
FMDV sub-genomic replicon and PK schematic. Schematic of the FMDV O1K sub-genomic replicon, showing both 5’ and 3’ untranslated regions (UTRs) together with the RNA structures present in these regions. IRES-driven translation produces a single polyprotein. Here, the structural proteins have been replaced with a green fluorescent protein (GFP) reporter gene upstream of the non-structural proteins 2A-3D. The PK encoding region is circled in yellow (A) and the interactions proposed in 1987 highlighted in hot-pink. Numbers indicate nucleotide positions after the poly-C-tract (B). Sequence alignment of the 4 PKs, with the interacting regions shown in hot-pink and invariant nucleotides represented by asterisk (C).

The FMDV genome (approximately 8.4 kb) consists of a single open reading frame flanked by 5′ and 3′ untranslated regions (UTRs), and a poly A tail. The 5′ UTR is covalently linked to a small protein primer, VPg (Fig 1A) [11]. The translated region encodes a polyprotein, which includes both structural and non-structural proteins. A viral protease, L^pro^, present at the N terminus of the polyprotein precedes the P1-2A region, which encodes the capsid structural proteins VP1, VP3, and VP0 (which is further processed to VP2 and VP4 during virus assembly), and the 2A non-structural protein [12]. The P2 and P3 regions encode the non-structural proteins 2B and 2C, and 3A, 3B _(1–3)_ (VPg), 3C^pro^ and 3D^pol^, respectively [13,14].

FMDV has the largest known 5′ UTR of the picornaviruses, comprising several highly structured regions and spanning approximately 1,300 nucleotides (nts). In contrast, the 5′ UTR from poliovirus comprises approximately 750 nt. The first 300–400 nts of the FMDV 5′ UTR (depending on virus isolate) are predicted to fold into a single large stem-loop termed the S-fragment. This is followed by a large poly-C tract of variable length (which can be up to 200 nts), a region containing two to four tandemly repeated PKs, the cis acting replication element (*cre*) and the internal ribosome entry site (IRES) [11,15,16]. Of these five structural domains, clearly defined functions have been ascribed to only two, the *cre* and IRES. The *cre* region is involved in templating uridylation of the RNA primer peptide, VPg (also known as 3B), and the IRES determines the initiation of translation of the viral polyprotein [17,18]. The roles of the S-fragment, the poly-C tract and the PKs in viral replication have not been fully elucidated, however, recent studies have shown that truncations to the S-fragment can affect the ability to control aspects of innate immune response to viral infection [19–21]. It has also recently been reported that viruses with a deletion within the PK region exhibited an attenuated phenotype in bovine but not in porcine cells, suggesting a role for the PKs in determining viral tropism [22].

The PKs were originally computationally predicted in 1987 using what is now known as the DotKnot program [16]. Two to four tandem repeats of a ~48 nts region containing a small stem-loop and downstream interaction site were predicted (Fig 1B). Due to the sequence similarity between the PKs (Fig 1C), it is speculated that these were formed by duplication events during viral replication, probably involving recombination. Between two and four PKs are predicted in different virus isolates but no strain has been identified with less than two PKs, emphasising their potential importance in the viral life cycle [23,24]. The presence of PKs has been reported in the 5′ UTR of only two other picornaviruses, encephalomyocarditis virus (EMCV) and equine rhinitis A virus (ERAV) [25,26]. However, in both cases the PKs are located at the 5′ side of the poly-C-tract, making their location in the FMDV genome unique.

More widely, PKs have been reported to have roles in several aspects of viral replication including RNA splicing (e.g. HIV and influenza), ribosomal frameshifting (e.g. coronaviruses) and RNase protection (e.g. Dengue virus) [27–31]. In the work reported here, the conserved RNA structure of FMDV PKs and the role of the predicted PKs in the FMDV life cycle was investigated.

## Materials and methods

### Cells lines

Baby hamster kidney cells (BHK-21) and Madin Darby bovine kidney (MDBK) cells were obtained from the ATCC (LGC Standard) and were maintained in Dulbecco’s modified Eagle’s Medium with glutamine (Sigma-Aldrich) supplemented with 10% foetal calf serum (FCS), 50 U/ml penicillin and 50 μg/ml streptomycin. Both cell lines are known to support replication of FMDV. BHK-21 cells are used for commercial FMD vaccine production while the MDBK cell line originates from a natural host of FMDV.

### Plasmid construction

The FMDV replicon plasmids, pRep-ptGFP, and the replication-defective polymerase mutant control, 3D-GNN, have been previously described [32].

To introduce mutations into the PK region, the pRep-ptGFP replicon plasmid was digested with *Spe*I and *Kpn*I and the resulting fragment inserted into a sub-cloning vector (pBluescript) to create the pBluescript PK. PKs 3 and 4 were removed by digestion with *Hind*III and *Aat*II and the fragment replaced by a sequence lacking PKs 3 and 4. PKs 2, 3 and 4 were deleted by PCR amplification using ΔPK 234 forward primer and FMDV 1331–1311 reverse primer, the resultant product was digested with *Hind*III and *Aat*II and ligated into the pBluescript PK vector. Complete PK deletion was achieved by introduction of an *Afl*II site at the 3′ end of the poly-C tract by PCR mutagenesis to create the sub-cloning vector, pBluescript C11, which lost all but 11 C residues during the manipulation procedure. This was then used to remove all the PKs by PCR mutagenesis using ΔPK 1234 forward primer and FMDV 1331–1311 reverse primer. The modified PK sequences were removed from the sub-cloning vectors and inserted into the pRep-ptGFP plasmid using *Nhe*I-HF and *Kpn*I-HF.

Mutations to disrupt PK structure were introduced using synthetic DNA by digestion with *Afl*II and *Aat*II and ligation into a similarly digested pBluescript PK vector. Mutations were then introduced into the replicon plasmid as described above.

To assess the effects of truncation of the poly-C-tract on replication the entire sequence was removed. This was performed by PCR mutagenesis using primers C0 *Spe*I, and FMDV 1331–1311 as forward and reverse primers respectively. The PCR product was digested with *Spe*I and *Kpn*I before ligation into a *Nhe*I and *Kpn*I digested *wt* pRep ptGFP replicon. Sequences of all primers are available upon request.

### *In vitro* transcription

*In vitro* transcription reactions for replicon assays were performed as described previously (26). Transcription reactions to produce large amounts of RNA for SHAPE analysis were performed with purified linear DNA. 1 μg of linearised DNA was used in a HiScribe T7 synthesis kit (NEB), before DNase treatment and purification using a PureLink RNA mini kit (Thermo Fisher).

### Computational prediction of PK structure

To define the conserved structure of PKs of FMDV field isolates, the full genome sequences of 118 FMDV isolates were obtained from GenBank (S1 Table). The FMDV isolates were chosen based on the variability of the region encoding the VP1 capsid protein, which is the most variable genomic region, in such a way that the dataset represented the currently known FMDV variability within all seven FMDV serotypes. The FMDV whole genome sequences were aligned using MAFFT G-INS-i algorithm [33–35], the genomic region encoding the PK 1–4 sequence was extracted and the multiple sequence alignment (MSA) re-aligned by the MAFFT L-INS-i algorithm. The regions of the MSA containing each individual PK (1–4) were isolated and subjected to PK prediction using a pAliKiss program implemented in Shapes Studio of the BiBiServ [36]. Covariance analysis of the pairings within stems was performed using RNAalifold program implemented in The ViennaRNA Package and the covariance analysis of the pairings which form the PK structures was determined manually. Individual PK structures were visualised using the Forna web server [37,38].

Predictions of PK structures located in the genome of the FMDV sub-genomic replicon were carried out using ShapeKnots program implemented in the RNAstructure v.6.2 package, with the structure of each PK encoding region being predicted individually [39–41]. Average SHAPE reactivity data obtained from four individual experiments (described below) acted as constraints for this computational analysis. Again, structures of PKs were visualised using the Forna web server [38]. RNA structure prediction of mutated PK sequences was carried out using the DotKnot algorithm [42–44].

### Selective 2′ hydroxyl acetylation analysed by primer extension (SHAPE) analysis

FMDV replicon RNA was prepared as above and a sample (12 pmol) was heated to 95°C for 2 minutes before cooling on ice. RNA folding buffer (100 mM HEPES, 66 mM MgCl_2_ and 100 mM NaCl) and RNase Out (Invitrogen) was added to the RNA and incubated at 37°C for 30 minutes. Once folded, RNA was treated with NMIA compound at a final concentration of 5 mM or DMSO as a negative control for 50 minutes at 37°C. Following incubation, modified RNA was precipitated with ethanol and resuspended in 10 μl 0.5 x TE buffer. Prior to use, the RNA integrity was assessed by gel electrophoresis.

Primer extension of NMIA modified RNA was performed by incubation of 5 μl of RNA with 6 μl of RNase free water and 1 μl of 10 μM Hex of FAM fluorescent primer (5′ GTCAGTTGGGGAAACTGC 3′). Primer binding was facilitated by heating the reaction to 85°C for 1 minute, 60°C for 10 minutes and 35°C for 10 minutes in a thermocycler. A reverse transcription master mix containing 4 μl of first strand buffer, 1 μl of 100 mM DTT, 0.5 μl of RNase Out, 1 μl of Superscript III (Invitrogen), 1 μl of 10 mM PCR dNTP mix (Promega) and 0.5 μl of RNase free water, was then added to the RNA/primer complex and extension carried out by incubation at 52°C for 30 minutes.

Post extension, cDNA:RNA hybrids were disassociated by incubation with 1 μl of 4M NaOH at 95°C for 3 minutes before neutralisation with 2 μl of 2 M HCl. Extended cDNA was precipitated with ethanol and resuspended in 40 μl of deionized formamide (Thermo Fisher). Sequencing ladders were made similarly using 6 pmol of RNA with the inclusion of 1 μl of 10 mM ddCTP in the reverse transcription mix and using a differentially labelled fluorescent primer (either Hex or FAM). Sequencing ladder (20 μl) was combined with NMIA or DMSO samples and dispatched on dry ice for capillary electrophoresis (Dundee DNA seq).

Capillary electropherograms were analysed using QuShape and reactivity overlaid onto the RNA structure using VARNA [45, 46]. Data shown are representative of 4 independent experiments, As in previous publications, nts with normalised SHAPE reactivity were shown as; 0–0.3, unreactive, 0.31–0.7, moderately reactive and >0.7, highly reactive [47].

### Replication assays

Replicon replication in all cell lines was assessed in 24-well plates with 0.5 μg/cm^2^ of RNA using Lipofectin transfection reagent (Life Technologies) as previously described [48]. BHK-21 or MDBK cells seeded into 24-well plates were allowed to adhere for 16 hours before transfection with 1 μg of replicon RNA using Lipofectin. Each transfection was performed in duplicate and experiments were biologically repeated. Replicon replication was assessed by live cell imaging using an IncuCyte Zoom Dual colour FLR, an automated phase-contrast and fluorescence microscope within a humidifying incubator. At hourly intervals up to 24 hours post transfection, images of each well were taken and used to count the number of ptGFP positive cells per well. We have previously shown that counting numbers of fluorescent cells or total fluorescence per well gave equivalent results. For clarity, the replication kinetics experiments were represented by data at 8 hours post transfection (hpt) when the expression of reporter from the wt FMDV replicon reached a maximum value [32].

Passaging in competition assays was performed by co-transfecting BHK-21 cells with *in vitro* transcribed competing replicon RNAs and harvesting total cell RNA at 8 hours post transfection using TRIzol reagent (Thermo Fisher Scientific). The harvested RNA was then purified using the Direct-zol RNA MiniPrep kit (Zymo Research) with on-column DNase I treatment and eluted in DEPC treated water. The purified passaged RNA (1 μg) was transfected onto naïve BHK-21 cells as above.

### Construction of recombinant viruses

Replicons used here are based on plasmid T7S3, which encodes a full length infectious copy of FMDV O1 Kaufbeuren. The reporter gene was removed from replicons by digestion with *Psi*I and *Xma*I restriction enzymes and replaced with the corresponding fragment from pT7S3 encoding the capsid proteins. Full length viral RNA was transcribed using a T7 MEGAscript kit (Thermo Fisher Scientific), DNase treated using TurboDNase (Thermo Fisher Scientific) and purified using a MEGAclear Transcription Clean-Up kit (Thermo Fisher Scientific). RNA quality and concentration were determined by denaturing agarose gel electrophoresis and Qubit RNA BR Assay Kit (Thermo Fisher Scientific).

### Virus recovery

BHK-21 cells were transfected in 25 cm^2^ flasks with 8 μg per flask of infectious clone-derived RNA using TransIT transfection reagent (Mirus) as described previously [14]. At full cytopathic effect (CPE) or 24 hours post-transfection (whichever was earlier) cell lysates were freeze-thawed and clarified by centrifugation. Clarified lysates were passaged onto naïve BHK-21 cells; this was continued for five rounds of passaging. Each time virus was harvested at full CPE or at 24 hours post infection if no CPE was observed.

### Sequencing of recovered virus

Recovered viruses at passage 4 were freeze-thawed and cellular debris removed by centrifugation. Total RNA was extracted from clarified supernatants and sequenced with an Illumina MiSeq (Illumina). Analysis of Illumina sequencing reads was conducted as described in [49].

### Plaque assay of recovered virus

Confluent BHK-21 cell monolayers were infected with 10-fold serial dilutions of virus stock, overlaid with Eagle overlay media supplemented with 5% tryptose phosphate broth solution (Sigma Aldrich), penicillin (100 units/ml) and streptomycin (100 μg/ml) (Sigma Aldrich) and 0.6% Indubiose (MP Biomedicals) and incubated for 48 hours at 37°C. Cells were fixed and stained with 1% (w/v) methylene blue in 10% (v/v) ethanol and 4% formaldehyde in PBS.

Fixed plaques were scanned, and images measured using a GNU Image Manipulation Program IMP (GIMP, available at https://www.gimp.org). For each plaque, horizontal and vertical diameter in pixels was taken and an average of these two values was calculated. All plaques per well were measured.

### Cell killing assays

Virus titres were determined by plaque assays. BHK-21 cells were seeded with 3 x 10^4^ cells/well in 96 well plates and allowed to settle overnight. Cell monolayers were inoculated with each rescued virus at MOI of 0.01 plaque forming units (PFU) for 1 hour, inoculum was removed and 150 μl of fresh GMEM (supplemented with 1% FCS) was added to each well. Appearance of CPE was monitored every 30 minutes using an Incucyte S3 live-cell analysis system (Essen BioScience).

### Flow cytometry assay

Triplicate cultures of BHK-21 cells in T25 flasks were transfected for 1 hour with 10 μg of *in vitro* transcribed full length viral RNA using Lipofectamine 2000. The transfection mix was replaced with fresh medium and after a further 3 hours, cells were released from the plastic using trypsin-EDTA and fixed in 4% paraformaldehyde for 40 minutes. Production of virus proteins was revealed by labelling the cells with anti-3A mAb 2C2 and AlexaFluor488 labelled anti-mouse secondary antibodies diluted 1 in 1000 and 1 in 200 respectively in 0.5% BSA in PBS blocking buffer (Melford). Labelled cells were analysed by the LSR Fortessa (BD Biosciences) using BD FACSDiva software. Data were exported as flow cytometry standard (FCS) files and were analysed in FlowJo 10. Levels of 3A expression were inferred from the signal from the fluorescent secondary antibodies present in each cell and resulted in a mean fluorescence intensity (MFI) value for a given population of cells. Virus positive/negative populations were identified based on levels of Alexa-488 fluorescence. Where distinct virus positive and negative populations were present, gates were drawn to separate these and selectively determine the mean fluorescent intensity (MFI) of the virus positive cells. Where no clear separate populations existed (wt with GuHCl, mock treatment and untreated cells) gates could not be drawn and therefore the total MFI was reported.

## Results

### Conserved structure prediction confirms PKs within FMDV genome

**Fig 2:**
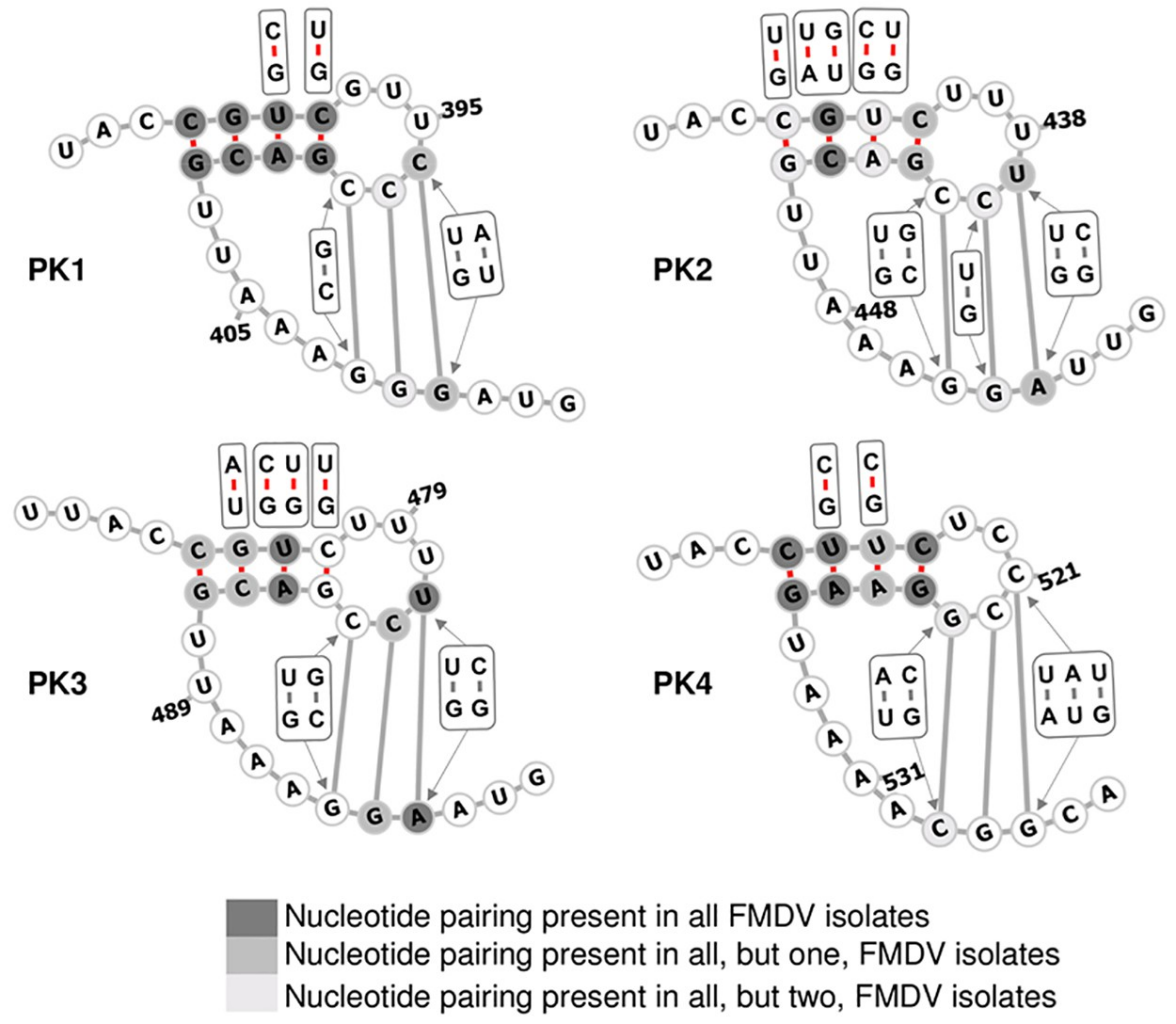
A schematic representation of conserved RNA structure formed by each pseudoknot region (PK 1–4) of FMDV. Genomic regions of 118 FMDV sequences representing all 7 serotypes and thought to form PKs were aligned. The regions of the multiple sequence alignment containing each individual PK (1–4) were isolated and subjected to PK prediction using a pAliKiss program implemented in Shapes Studio of the BiBiServ. Covariance analysis of the pairings within stems was performed using RNAalifold program implemented in The ViennaRNA Package. The covariance analysis of the parings which form the PK was determined manually. PK structures were visualised using the Forna web server. Conservation of a nucleotide pairing was presented by three shades of grey, with dark shade showing nucleotide pairings which occurred in all FMDV isolates, medium shade showing nucleotide pairings which occurred in all but one FMDV isolates, and light shade showing lack of a nucleotide pairing in two FMDV isolates. Nucleotide pairings which were absent in three or more FMDV isolates were left unmarked. Where detected, alternative nucleotide pairing types are shown in squares next to their nucleotide pairing position. Numbers represent nucleotide positions corresponding to the sequence of A/Brazil/1979 isolate (GenBank accession number AY593788). S2 Table specifies details represented graphically in the figure legend. Table 1 details the extent of conservation for each pairing forming the PKs.

The PKs within the FMDV genome were predicted in 1987 [16] (Fig 1), well before the advances of high throughput sequencing resulted in hundreds of full genome FMDV sequences being available on GenBank. Therefore, we decided to use the new data to investigate the conservation of these structures. Using a dataset of 118 FMDV field isolates, representing the currently known FMDV genomic variability across all seven serotypes (S1 Table), we predicted a conserved structure for each PK (1–4) individually and performed a covariance analysis to show the extent of nucleotide pairing conservation. For each PK, the stem-loop component of the structure comprised four nucleotide pairings, while the PK structure was stabilised by three further nucleotide pairings (Fig 2). All four PKs showed strong conservation of the stem-loop region with evidence of covariance that preserves the structures (Fig 2), suggesting that evolutionary constraints maintain these structures despite sequence variation. Although to a lower extent than within the stem-loop regions, the pairings forming the PK structures were also conserved, with evidence of covariance to maintain these structures (Fig 2 and Table 1). For most nucleotide positions involved in formation of the PKs, more than 90% of isolates formed a pairing (Table 1).

**Table 1:** Extent of pairing conservation forming each PK.

Pseudoknot	Number of isolates containing the PK^a^	Nucleotide positions forming a PK pairing^b^	Number of compatible pairs	Type of compatible pairs^c^	Number of isolates containing incompatible pair^d^
PK1	62/118^e^	396–410	3	C-G, U-G, A-U	1/62
		397–409	1	C-G	2/62
		398–408	2	G-C, C-G	6/62
PK2	112/118	439–453	3	U-G, C-G, U-A	1/112
		440–452	2	C-G, U-G	2/112
		441–451	3	C-G, G-C, U-G	3/112
PK3	118/118	481–495	3	C-G, U-G, U-A	0/118
		482–494	1	C-G	1/118
		483–493	3	C-G, G-C, U-G	5/118
PK4	117/118	521–535	4	C-G, U-A, A-U, U-G	12/117
		522–534	1	C-G	15/117
		523–533	3	G-C, C-G, A-U	2/117

^*a*^Number of FMDV isolates, out of 118, containing a complete PK sequence.

^*b*^Numbers represent nucleotide positions corresponding to the sequence of A/Brazil/1979 isolate (GenBank accession number AY593788).

^*c*^There are six possible compatible pairs: C-G, G-C, A-U, U-A, G-U and U-G.

^*d*^Number of FMDV isolates containing an incompatible pair (i.e., did not form a pairing) from the pool of sequences which contained a complete PK.

^*e*^Since PK1 is positioned directly downstream of poly-C tract, which is difficult to sequence, some of the apparent deletions in the PK1 region may be due to interrupted sequencing through the poly-C tract.

### SHAPE-guided prediction of PK structures

Many of our previous studies on replication involved the use of replicons. These allow replication to be studied without consideration of the rest of the lifecycle and can therefore be useful in dissecting the viral lifecycle. The replicons were generated by replacing the structural coding region of the viral genome with a fluorescent reporter gene (green fluorescent protein (GFP) from *Ptilosarcus gurneyi* (ptGFP) or mCherry). This allows real-time analysis of replicon replication through monitoring of fluorescence and also permits the use of such modified genomes outside of the high containment facilities required to study full-length FMDV genomes [32,50]. Here, we used a sub-genomic replicon based on FMDV O1K that we have employed previously in replication assays and which is predicted to contain 4 PKs. Before studying the functions of the predicted PKs, we verified their formation within the replicon RNA using SHAPE.

**Fig 3:**
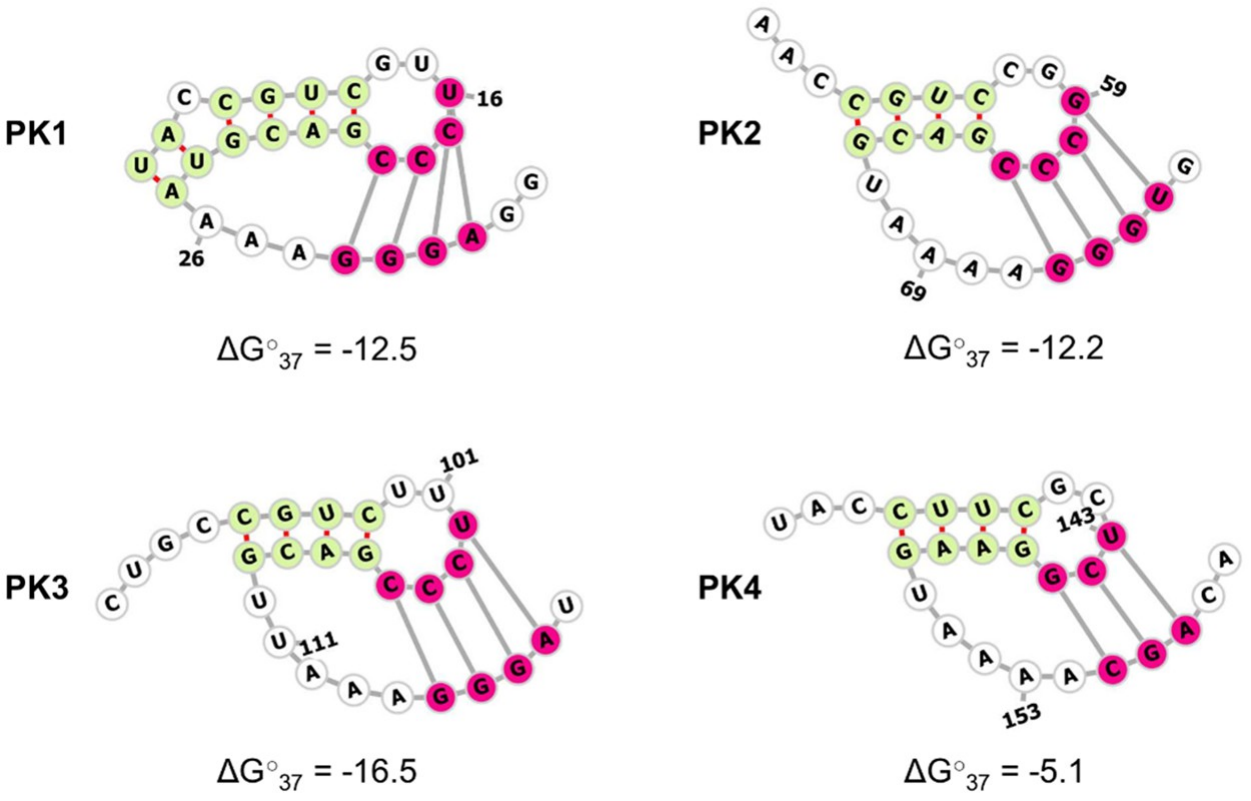
Schematic representation of RNA structure formed by each PK region (PK1-4) of the FMDV sub-genomic replicon. FMDV replicon RNA was subjected to selective 2′ hydroxyl acetylation analysed by primer extension (SHAPE), with the primer extension covering the first 239 nucleotides downstream of the poly-C tract. The averaged results of four independent experiments were used as constraints for computational RNA structure prediction using ShapeKnots program implemented in the RNAstructure v.6.2 package, with the structure of each PK encoding region being predicted individually. Nucleotide pairings forming a stem-loop are highlighted in pale green, while nucleotide pairings forming the PK are highlighted in hot pink. Minimum energy values (ΔG○37 = [kcal/mol]) for each PK are stated underneath the corresponding structures. Using this methodology, no alternative structures were predicted. Numbers represent nucleotide positions corresponding to the FMDV sub-genomic sequence downstream of the poly-C tract.

SHAPE primer extension was used to cover the first 239 nts downstream of the poly-C tract. The data from four independent SHAPE experiments (S2 Table) were used to constrain computational PK predictions using the ShapeKnots program implemented in the RNA structure v.6.2 package [39–41], with each PK1-4 being predicted independently. All four regions encoding the predicted PKs folded into H-type PK structures, with PK1-3 forming four nucleotide pairings between the nucleotides forming the hairpin loop (i.e., the unpaired loop at the top of the stem-loop structure) and the nucleotides positioned downstream of the stem-loop structure, while PK4 formed three such pairings (Fig 3). Using this methodology, no alternative structures were predicted for each of the PK genomic regions. This result, derived from a combination of physical data and prediction, refined the earlier simplistic predictions, and provided increased confidence in the presence of the PKs.

### A single PK is sufficient for efficient replication

**Fig 4:**
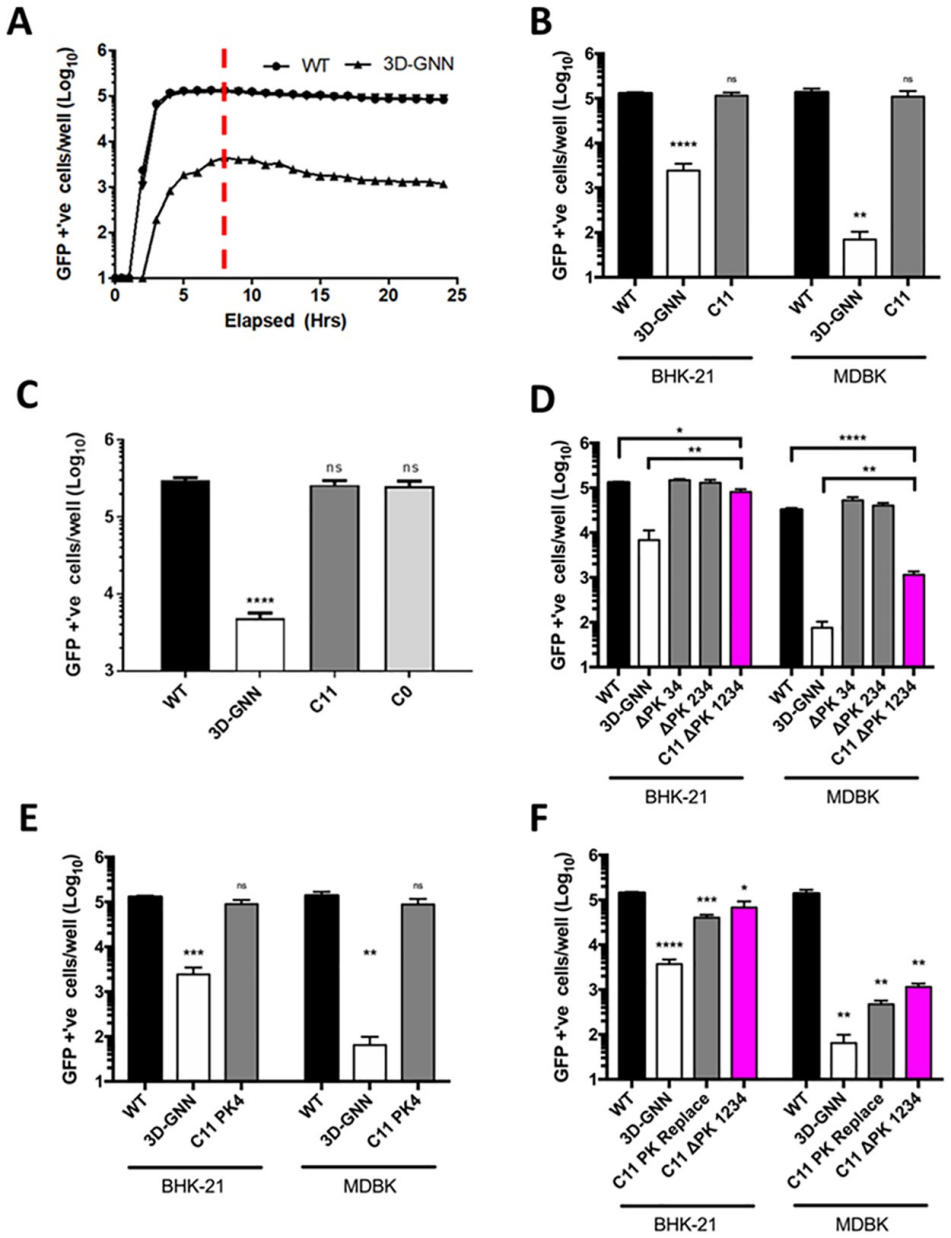
The poly-C-tract is dispensable and only one PK is required for near *wt* replication. Replicons were transfected into cell lines, and replication monitored by reporter gene expression using an IncuCyte Zoom. The *wt* and 3D-GNN constructs were transfected into BHK-21 cells and replication monitored hourly to observe peak reporter expression (A). *wt*, 3D-GNN and a replicon with a truncated poly-C-tract (C11) were transfected into BHK-21 and MDBK cells (B). A replicon with entire poly-C-tract removed (C0) was transfected alongside *wt*, 3D-GNN and C11 replicons into BHK-21 cells (C). Replicons with sequentially deleted PKs (ΔPK 34, ΔPK 234 and C11 ΔPK 1234) were assayed for replication in BHK-21 cells or MDBK cells (D). Replication of replicon with PK 4 as the sole remaining PK (C11 PK 4) transfected into MDBK or BHK-21 cells (E). The PK region was deleted and replaced with a scrambled sequence (C11 PK Replace). This construct was transfected into BHK-21 and MDBK cells alongside *wt*, 3D-GNN and C11 ΔPK 1234 replicons (F). All replication assays were measured by counting the number of GFP positive cells per well using the IncuCyte Zoom with data shown at 8 hours post-transfection. Error bars shown are calculated by SEM, n = 3. Significance is shown compared to the *wt* (A, B, C, E, F) or the *wt* and 3D-GNN (D) * P < 0.05, ** P < 0.01, *** P < 0.001, **** P < 0.0001. ns = not significant.

The PK sequences are thought to have arisen by duplication events, probably resulting from recombination during genome replication. Their high sequence similarity (Fig 1C) makes it difficult to ascertain which individual copies remain in virus strains containing fewer than four PKs. To investigate the minimum number of PK structures essential for FMDV genome replication, we deleted individual PKs sequentially from the 3′ end of the PK region, as graphically represented in S2 Fig. This was straightforward for PKs 2, 3 and 4. However, to allow complete removal of all PKs, it was necessary to insert an *Afl*II restriction site into the ptGFP replicon plasmid at the 5′ end of the PK region. This manipulation resulted in a truncation of the poly-C tract from a length undeterminable by sequencing to 11 cytosine residues. Therefore, we first investigated the C11 replicon alongside a wild-type (*wt)* and the 3D-GNN replicon containing an inactive polymerase [32]. RNA was transfected into BHK-21, a cell line commonly used to study FMDV replication, or bovine MDBK cells and data are shown at 8 hours post-transfection to include the maximum amount of *wt* replication. The 3D-GNN replicon allowed for monitoring of level of input translation in the absence of replication (Fig 4A). The data show that the C11 replicon replicated similar to the *wt* in both cell types, which is consistent with previous studies (Fig 4B) [51]. To confirm that there was no negative effect of poly-C tract truncation on replication of the replicon, we removed the entire poly-C tract (to generate C0). Fig 4C shows that this complete removal of the poly-C tract had no effect on replication of the replicon in BHK-21 cells. These results suggest that the poly-C tract is not required to maintain the structure or function in the PK region in the replicon system.

Following transfection of BHK and MDBK cells, replicons lacking PK 3 and 4, or PK 2, 3 and 4 (termed ΔPK 34 and ΔPK 234, respectively) replicated at similar levels to the *wt* replicon (Fig 4D and S1 Fig). However, a replicon with all four PKs deleted (C11 ΔPK 1234) showed a significant (~4 fold) reduction in replication in BHK-21 cells, and a larger reduction (28-fold) in MDBK cells. Interestingly, the GFP signal of the C11 ΔPK 1234 replicon was still significantly above the 3D-GNN negative control (11-fold), showing that this replicon was capable of replication. This suggests that the PKs are not essential for FMDV replicon replication, but that the presence of at least one PK is required to achieve near *wt* replication levels. Analysis of the full time course of replication indicated no change in replication kinetics and all constructs reached maximum replication by 8 hours post transfection (S1 Fig).

In the above experiments, the ΔPK 234 construct (which contained only PK1) achieved near *wt* replication levels, which suggests that only one PK is required for viral RNA (vRNA) replication. To investigate this further, we determined whether PK1 was essential for FMDV replicon replication or whether this could be replaced with another PK. As PK4 differed most from PK1 (PK1, PK2 and PK3 share high levels of sequence similarity, Fig 1C), we generated a replicon (C11 PK4) which had PK4 as the only PK and determined whether there were any functional consequences for replication in BHK or MDBK cells. Replication of the C11 PK4 construct showed similar levels of replication to *wt* with no significant difference in either cell line (Fig 4E). This observation suggests that there is no observable difference between PK1 and PK4 and that the individual PKs provide similar functions during replicon replication. Although we cannot totally rule out any long-range interactions, the similar levels of replication observed for constructs with either only PK1 or PK4 suggests that the PKs function as independent structures, which do not require interaction between each other.

The above observations suggest that a single PK region is sufficient to achieve near *wt* replication of the FMDV replicon. To investigate if PK structure is important for replication, or whether this region simply acts as a spacer between other elements within the 5′ UTR, we replaced the PK region with a scrambled version of the sequence. This artificial sequence contained the same nucleotides, but with their positions randomised, and analysis using DotKnot PK predicted no PK structures. This sequence was created by gene string and cloned into the replicon using the *Afl*II site to create ‘C11 PK Replace’. This replicon was transfected in parallel with *wt*, 3D-GNN and C11 ΔPK 1234 replicons into either BHK-21 or MDBK cells (Fig 4F). Replacement of the PK region with the scrambled sequence was more deleterious to replicon replication than having no PKs at all (i.e. C11 ΔPK 1234), suggesting that the introduction of this artificial sequence may result in inappropriate interactions with other components of the 5′ UTR.

### Function of the PKs in replication is dependent on downstream interactions and orientation

Since removal of all four PKs resulted in a significant decrease in replicon replication, but a single PK was sufficient to maintain near *wt* levels of replication, we further explored the minimal requirements to maintain this level of replication. As near *wt* levels of replication were observed when only one PK was present, all further mutagenesis was performed in a C11 replicon plasmid containing only PK1.

**Fig 5:**
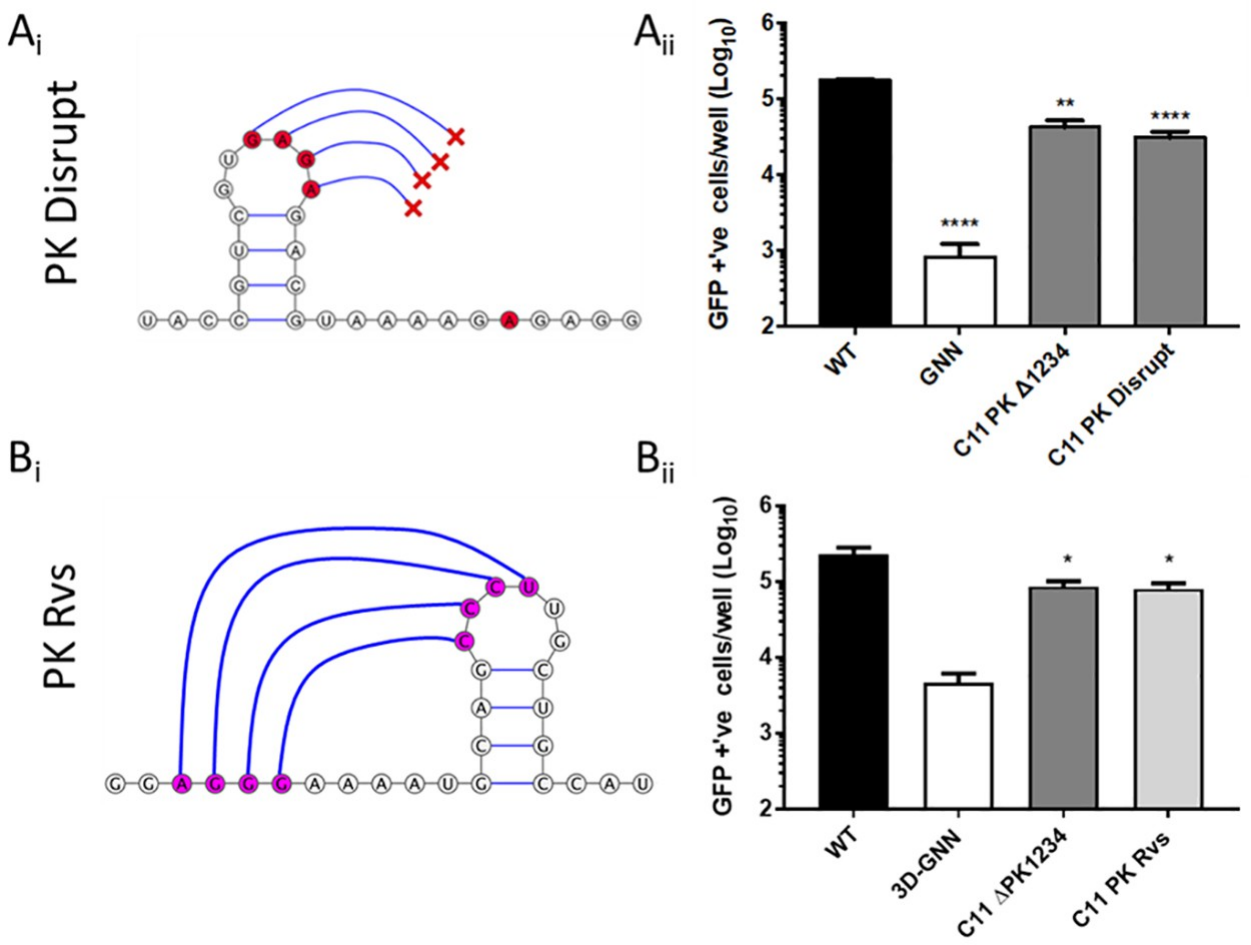
PK structure is essential for function. Cartoon representations of the PK disrupting mutations made to PK 1, where nucleotides in the bulge of the stem loop and the predicted interacting region downstream were mutated to prevent base-pairing ‘PK disrupt’ (A_i_). Replication of PK disrupt mutant was measured by transfection of RNA into BHK-21 cells and recording fluorescence at 8 hours post-transfection. Significance is shown comparing the replication of mutants against the *wt* (A_ii_). Visual representation of the reversal of the nucleotide sequence of PK1 to create the C11 PK Rvs (reverse) construct (B_i_). Replication of PK Rvs was compared to *wt*, 3D-GNN and C11 ΔPK 1234 controls as in A_ii_ (B_ii_). Significance shown is compared to *wt* replicon. Error bars are calculated by SEM, n = 3, * P < 0.05, ** P < 0.01 **** P < 0.0001.

Substitutions (shown in red in Fig 5) were designed to interrupt base pairing by creating GAGA motifs in both the stem-loop and downstream nucleotides, thereby abrogating the possibility of forming the predicted PK1 structure, as confirmed using DotKnot (Fig 5A_i_). These mutations significantly reduced the replication of the mutated replicon (C11 PK disrupt) to the level of the replicon containing no PKs (Fig 5A_ii_). In addition, the orientation of PK 1 was reversed by “flipping” the nucleotide sequence to potentially facilitate hybridisation of the loop with upstream rather than downstream sequences (PK Rvs (reverse)) (Fig 5B_i_). Changing the orientation of the PK in this way reduced replicon replication to a similar level seen in the absence of PKs (Fig 5B_ii_).

### Multiple PKs confer a competitive replicative advantage

The deletion studies above suggested that removal of up to three of the four predicted PKs had only a small effect on replicon replication, whereas deletion of all four was clearly detrimental, although the level of replication was above the GNN control. To investigate whether multiple PKs conferred more subtle advantages for replication than were evident from single round transfection experiments, we carried out sequential passages of replicon RNAs following transfection of the PK deleted forms in competition with a *wt* replicon. Different reporter genes (ptGFP or mCherry) were used to distinguish the competing replicons, in a similar way to that reported in [52].

The wt replicon or the ΔPK 34, ΔPK 234 and C11 ΔPK 1234 replicons (which all encode ptGFP) were co-transfected into BHK-21 cells with either a *wt* mCherry replicon or yeast tRNA as a non-specific carrier control. Replication of each of the co-transfected replicons was compared by following ptGFP and mCherry expression over three sequential passages. Passaging was achieved by harvesting total RNA using Trizol-reagent 8 hours post-transfection. Harvested RNA was purified and then re-transfected into naïve BHK-21 cells.

**Fig 6:**
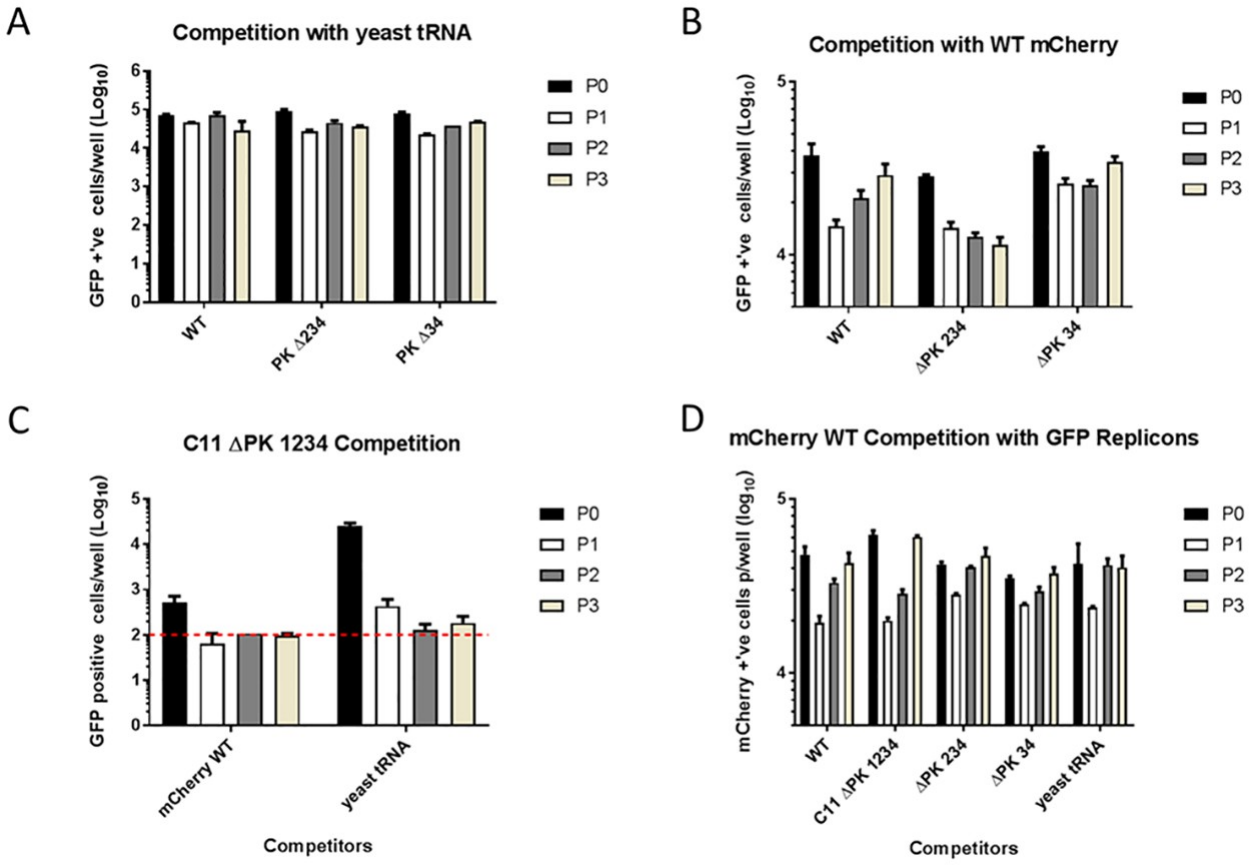
Presence of at least two PKs within FMDV replicon provides a replicative advantage in co-transfection competition experiments. BHK-21 cells were co-transfected with *wt*, C11 ΔPK 1234, ΔPK 234 or ΔPK 34 ptGFP replicon RNA and either a *wt* mCherry replicon or yeast tRNA non-specific carrier control. After ~1 cycle of replication (i.e 8 hours post-transfection, total RNA was harvested and re-transfected into naïve BHK-21 cells. Figure shows serial passage of ptGFP *wt*, ΔPK 234 or ΔPK 34 replicons with yeast tRNA non-specific carrier (A). Serial passage of *wt*, ΔPK 234 or ΔPK 34 ptGFP replicons in competition with a *wt* mCherry replicon (B). Serial passage of the C11 ΔPK 1234 replicon when co-transfected with either the *wt* mCherry replicon or yeast tRNA non-specific carrier control (C). Replication of the *wt* mCherry replicon when co-transfected with ptGFP *wt*, C11 ΔPK 1234, ΔPK 234 or ΔPK 34 ptGFP replicons (D). Data from three independent experiments is presented across the four panels, for clarity. All replication is shown at 8 hours post transfection over 3 sequential passages, as measured by an IncuCyte Zoom (n = 3). Initial transfection (P0), sequential passages (P1-3). Statistical comparison of competing replicons can be seen in S3 Table.

As expected, the *wt*, ΔPK 34 or ΔPK 234 replicons behaved similarly when passaged following co-transfection with yeast tRNA as non-specific control (Fig 6A). Furthermore, when PK mutants were co-transfected with a *wt* replicon, the GFP signal produced by the ΔPK 34 replicon was comparable to that of the *wt* after three passages, suggesting no clear competitive advantage of four PKs over two (Fig 6B). It should be noted that for both *wt* and ΔPK 34, there was a reduction in replication after the first passage but recovery to near that of the original transfection by the third passage. The initial drop in replication could be due to the first round being reliant on the T7 RNA transcript, whereas the passaged samples use genuine replicated RNA. The latter is probably a more efficient template, as it includes covalently bound VPg, for example, and hence expands on sequential passages. When the ΔPK 234 replicon was co-transfected with the *wt* mCherry replicon a similar drop in replication of both was seen in passage 1, but this decline in ΔPK 234 replicon signal continued in subsequent passages (Fig 6B and 6D). However, no decrease was observed when ΔPK 234 was co-transfected with yeast tRNA (Fig 6A). It thus appears that replicons with a single PK are at a competitive disadvantage compared to those with two or more.

Co-transfection of the C11 ΔPK 1234 replicon with the *wt* mCherry replicon resulted in greatly reduced replication when compared to corresponding co-transfection with yeast tRNA control. By passage one, the ptGFP signal of the C11 ΔPK 1234 had declined to background level, suggesting that this replicon was out-competed (Fig 6C). Although the initial replication of C11 ΔPK 1234 was greater when co-transfected with yeast tRNA than when in competition with *wt* mCherry replicon, the ptGFP signal was reduced at passage two and was at background level by passage three (Fig 6C). As a control, we demonstrated that replication of the mCherry *wt* replicon was not influenced by co-transfection with any of the ptGFP constructs (Fig 6D).

Together, these data suggest that the minor replicative advantage conferred by multiple PKs is quickly compounded over multiple replication cycles and at least partially explain why genomes of FMDV field isolates contain at least two PKs (S3 Fig.).

### The PKs are essential for the production of infectious virus

Since we demonstrated that constructs lacking all PKs could replicate and that replicons with reduced numbers of PKs appeared to be at a competitive disadvantage compared to the *wt* construct, we investigated the consequences of PK manipulation on the production of infectious virus. The ΔPK 34, ΔPK 234 and C11 ΔPK 1234 replicons were converted into FMDV infectious copy (IC) plasmids by replacing the sequence encoding ptGFP with the coding sequence of the O1K structural proteins. vRNA transcripts derived from the modified IC plasmids were transfected into BHK-21 cells in parallel with vRNA transcribed from *wt* O1K IC plasmid as a positive control and passaged 5 times by transferring the cell supernatants at full cytopathic effect (CPE) or at 24 hours post-transfection onto naïve BHK-21 cells. At passage 4, the recovered virus was harvested and sequenced to check for compensatory or reversion mutations.

**Fig 7:**
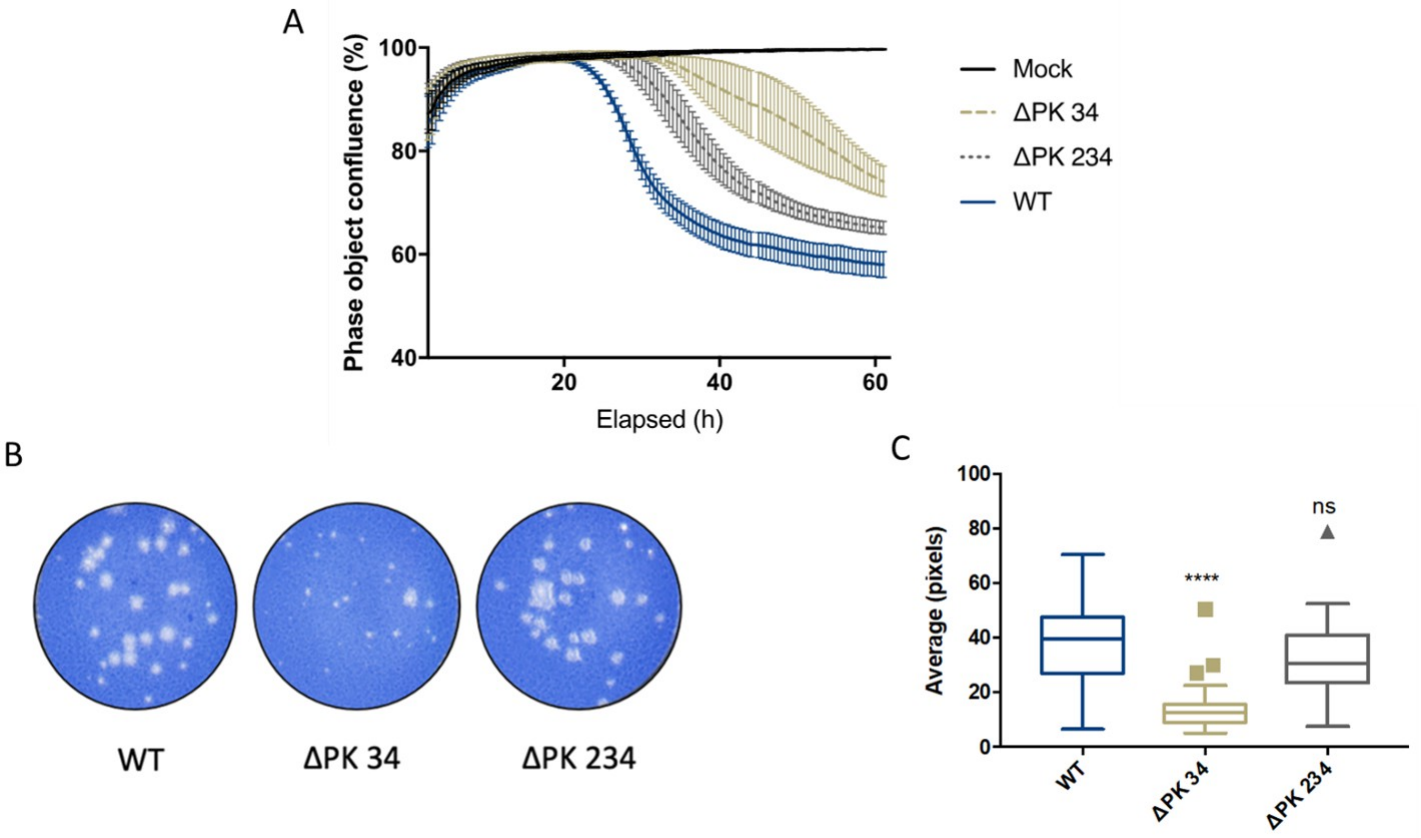
Recovered virus showed a delay in rate of CPE and a small plaque phenotype. BHK-21 cells were infected with *wt*, ΔPK 34 and ΔPK 234 virus at a MOI of 0.01, alongside a mock infected control and cell confluence (shown as phase object confluence %) monitored every half an hour for 62 hours using an IncuCyte Zoom (A). Representative plaque assay of BHK-21 cells infected with *wt*, ΔPK 34 and ΔPK 234 viruses. Virus recovered following 5 passages was used to infect BHK-21 cells, which were fixed and stained 48 hours post infection (B). Virus plaques were imaged and size of plaques measured using GIMP, all plaques per well were counted, additional wells were used until a minimum plaque count of 40 was reached. Significance is shown compared to the *wt*; box and whisker plots were made using the Tukey method (C), **** P < 0.0001, ns = not significant.

Transfection of the vRNAs for the *wt*, C11, ΔPK 34 and ΔPK 234 viruses generated infectious virus with no alteration to input sequence. However, C11 ΔPK 1234, which replicated (albeit to a lesser degree) as a replicon (Fig 2), produced no recoverable infectious virus (Table 2). Interestingly, there were differences noted in both the rate of development of CPE and plaque size of ΔPK 34 and ΔPK 234 when compared to the *wt* O1K virus. Rate of development of CPE was measured by infecting BHK-21 cells with a known MOI (0.01 PFU) of recovered virus and then monitoring cells for signs of CPE (shown as a decrease in cell confluence) as measured via IncuCyte S3 (Fig 7A). Both ΔPK 34 and ΔPK 234 showed delayed onset of CPE compared to the *wt* control. Surprisingly, the time to initial onset of CPE post-infection was 22 hours for the *wt* control compared to 39 hours for ΔPK 34 but only 29 hours for ΔPK 234. This mirrored data from plaque assays (Fig 7B and 7C), in which ΔPK 34 produced significantly smaller plaques compared to the *wt* control (average of 13.8 pixels compared to 37.4 pixels), however, the plaques produced by ΔPK 234 were not significantly different to *wt* (average 31.9 pixels).

**Table 2:** Virus could not be recovered when all PKs are deleted. Genomes of FMDV *wt* and modified FMDV sub-genomic replicons were converted into infectious viruses by swapping the GFP reporter gene with sequence encoding capsid of O1K FMDV infectious copy clone. RNA transcribed from these *wt* or modified FMDV genome clones was transfected into BHK-21 cells and appearance of CPE observed over 5 sequential passages. At the 4th passage virus was harvested and sequenced to observe for any changes within the sequence. Presence of CPE indicated with ‘Y’ while ‘N’ represents no CPE seen.

Recovered Virus	Appearance of CPE				Sequence of Rescued Virus
	Passage 1	Passage 2	Passage 3	Passage 4	
WT	Y	Y	Y	Y	No Change
C11	Y	Y	Y	Y	No Change
ΔPK34	Y	Y	Y	Y	No Change
ΔPK234	Y	Y	Y	Y	No Change
ΔPK1234	N	N	N	N	N/A

**Fig 8:**
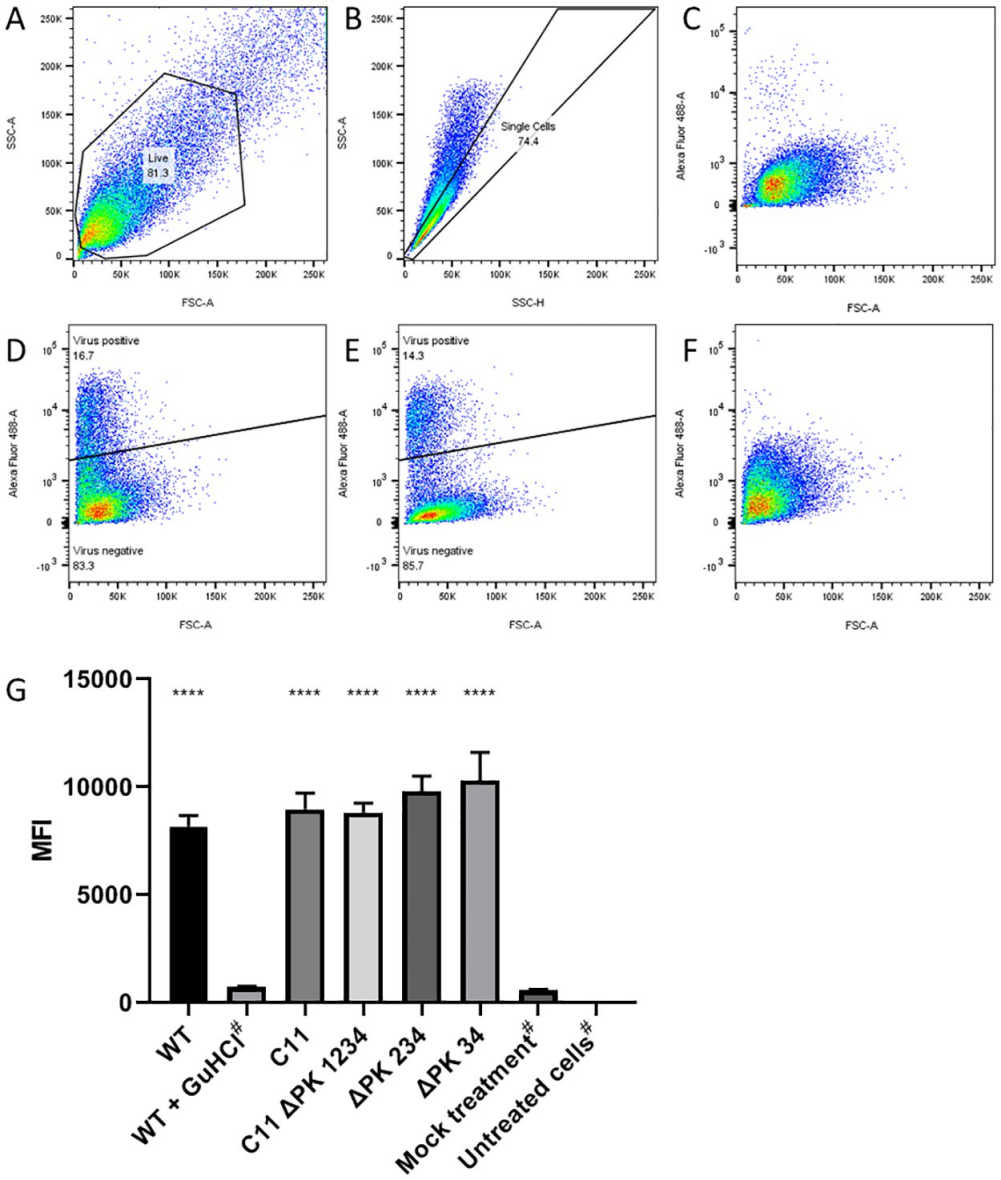
PKs are not essential for viral genome replication. BHK-21 cells were transfected with RNA transcribed from infectious clones of *wt*, ΔPK 34, ΔPK 234 and C11 ΔPK 1234. Non-transfected cells or cells transfected with *wt* but treated with 3 mM GuHCl to inhibit replication were used as negative controls. Cells were harvested, fixed, and labelled using an anti-3A antibody and fluorescent anti-mouse secondary before separation by flow cytometry. Representative images are shown here for the live cell gate from the *wt* virus transfection (A), the single cell gate from the *wt* virus transfection (B) and the relative fluorescence of the cells with mock antibody treatment (C), *wt* virus (D), C11 ΔPK 1234 virus (E) and *wt* virus transfection with 3 mM GuHCl (F). The experiment was performed in triplicate and the MFI values for each condition were calculated (G). Where no clear virus-positive population was evident, virus positive gates could not be drawn and therefore the total MFI has been reported (marked with #). The error bars represent the SEM. Significance is shown compared to the *wt* plus 3 mM GuHCl control (**** P < 0.0001).

Since C11 ΔPK 1234 produced no infectious virus, the ability of the full-length genome lacking PKs to replicate was investigated. BHK-21 cells were transfected with the same vRNA transcripts as above alongside controls; mock-transfected cells and/or cells transfected with *wt* and treated with 3 mM GuHCl (a replication inhibitor) as negative controls. Six hours post-transfection, cells were harvested, fixed, and labelled with an anti-3A antibody and fluorescent secondary antibody. Cells were then analysed using flow cytometry and the anti-3A antibody signal was used as an indirect measure of genome replication (Fig 8). The results were similar to those of the replicon experiments and showed that all the modified virus genomes could replicate. The inability of the C11 ΔPK 1234 genome to support production of infectious virus despite being able to replicate after transfection into cells, is consistent with a requirement for an RNA sequence within the PK region for virus assembly and supportive of a predicted packaging sequence previously reported in this region [53].

## Discussion

The 5′ UTR of FMDV is unique amongst picornaviruses due to its large size, with the presence of multiple RNA elements, some of which still have unknown function. One of these is a series of predicted PKs varying in number from 2–4 (depending on virus strain) located downstream of the poly-C tract. Due to the sequence similarity between PK segments, it is difficult to ascertain which individual PKs remain in strains with fewer than 4 PKs. In this study, we manipulated the PK regions to help understand their role in the viral life cycle and showed a role in genome replication dependent on sequence, structure, and orientation. Conserved structure prediction with covariance analysis supported the presence of PK structures [16]. Although our FMDV PK structure predictions show fewer parings (three instead of four) than were previously proposed for PKs 1 and 3, the strong conservation of these pairings among 118 FMDV isolates representing all seven FMDV serotypes suggests that the originally proposed PKs are conserved structural features of the FMDV genome. Additionally, combining SHAPE experiments with such computational prediction confirmed that the FMDV sub-genomic replicon used in this study contained four PKs. PK structure predictions were also supported by observation of the detrimental effects on replication of substituting nucleotides predicted to form key interactions between the loop and downstream sequences of PK1 and so disrupting the potential for PK formation.

It is likely that each of the PKs is functionally competent as no differences in replication were detected between replicons containing a single copy of PK1 or PK4. This observation is consistent with a previous report of deletion of PK1, along with the poly-C tract, which had no adverse effect on viral replication [51]. These data also support our findings that the truncation of the poly-C tract had no effect on replicon replication in the cell lines tested. As has been described with Mengo virus, it is possible that the poly-C tract has an essential role in other aspects of the viral lifecycle e.g. which cannot be recapitulated in a standard tissue culture system.

Although all FMDV isolates sequenced to date contain at least two PKs, replicon and recombinant viruses containing a single PK were still replication-competent. Moreover, replicons lacking all PKs were replication competent, albeit were less fit than the *wt* replicon. The effect on replication of deleting the PKs was more pronounced in MDBK cells compared to BHK-21 cells, this may reflect differential binding of bovine and hamster cellular proteins to PKs and is consistent with previous reports suggesting a role of PKs in determining host cell specificity [22]. Alternatively, these data may suggest involvement of the PK region in manipulating cellular innate anti-viral responses, which are known to be more active in MDBK cells than BHK-21 cells [54–56]. Viruses recovered from genomes with reduced numbers of PKs were slower growing and produced smaller plaques. However, recovered viruses with a single PK grew faster and produced larger plaques in BHK-21 cells than those with two PKs, for unknown reasons.

The presence of at least two predicted PKs in all viral isolates sequenced so far suggests that multiple PKs confer a competitive advantage in replication. Here, we showed by sequential competitive passage that replicons containing at least two PKs were maintained at a level similar to *wt*, but replicons containing only one PK showed a persistent decline in replication. These data therefore indicate a competitive advantage in replication provided by more than one PK but do not explain the presence of up to four PKs in field isolates (S3 Fig.). An additional advantage might be seen in cells closer to the natural host than BHK-21 or, alternatively, stochastic variation or subtle effects of host range may be responsible. Our study uses a simplistic model of viral replication, which allows us to dissect RNA replication from other stages in the viral life cycle, however, it does not mirror evolutionary pressures and bottlenecks of natural infection. Further studies are required to assess involvement of PKs in a natural infection.

Surprisingly, although removal of all four PKs resulted in a consistent decrease in replicon replication, the same was not observed for viral genomes lacking the PKs, which appeared to replicate normally (in terms of 3A production). We ascribe this apparent discrepancy to the different methods used to assay replication of replicons or full-length viral genomes. More important, however, is the observation that while genome replication still occurs upon removal of all four PKs in both replicon and viral systems, no infectious virus was produced. This demonstrates that multiple PKs are not essential to support genome replication but are essential for the formation of infectious virus. This suggests that the PKs may be required for virion assembly, in support of our previously published evidence for the presence of a packaging signal in this region [53].

In conclusion, our results suggest that the FMDV PK region has both essential and more subtle roles in genome replication. Further work is underway to investigate the potential role(s) of PKs FMDV genome packaging.

## Supporting information

S1 Table FMDV sequences obtained from GenBank. (DOCX)

S2 Table SHAPE reactivity data. (DOCX)

S3 Table Statistical comparison of all competing FMDV replicons across passage 0–3 (P0-3) as presented in Fig 6. * P < 0.05, ** P < 0.01, *** P < 0.001 **** P < 0.0001, ns = not significant.
(DOCX)

S1 Fig Replicon replication over time. Replication of WT, 3D-GNN, ΔPK 34, ΔPK 234 and C11 ΔPK 1234 in MDBK and BHK-21 cells (A and B, respectively). GFP expression was monitored hourly for 12 hours using an IncuCyte Zoom. (n = 3), error bars represent SEM.
(TIF)

S2 Fig Cartoon representations of the constructs used to generate the data shown in Fig 4. The length of poly-C-tract is represented as PC_n_T for *wt* and PC_11_T for truncated replicons.
(TIF)

S3 Fig Number of FMDV isolates containing specific PK arrangements. Genomic regions containing PKs of 118 FMDV isolates representing all 7 serotypes were aligned as described for Fig 2. The number of isolates containing specific PK arrangements (no PK; only a single PK present: PK1, PK2, PK3 or PK4; only two PKs present: PK12, PK23, PK34, PK13, PK14, PK24; only three PKs present: PK123, PK234, PK124, PK134; or all PKs present PK1234) was determined and visualised as number of isolates for each possible PK arrangement. Due to difficulty of sequencing through the poly-C tract, it is likely, at least for some of the isolates, that the lack of PK1 (which lies directly downstream of the poly-C tract) is a result of sequencing error.
(TIF)
