# Supplementary material for: The RNA pseudoknots in foot-and-mouth disease virus are dispensable for genome replication, but essential for the production of infectious virus
Source: PLoS Pathog. 2022 Jun 6;18(6):e1010589. doi: 10.1371/journal.ppat.1010589 (PMC9203018; doi:10.1371/journal.ppat.1010589)
Supplement: S1 Table — (DOCX) [file ppat.1010589.s001.docx]

**S1 Table**

| **Serotype:** | **Number of isolates:** | **GenBank accession numbers:** |
| --- | --- | --- |
| A | 19 | AY593788, MH053305, JF749843, HM854024, HQ832580, MH053306, KM268896, AY593802, KJ608371, MH053307, AY593751, AY593754, AY593761, AY593764, AY593766, AY593767, HM854022, AY593791, AY593794 |
| Asia 1 | 12 | AY593795, AY687334, DQ533483, DQ989306, DQ989315, DQ989319, EF149010, EF614458, HQ632774, JF739177, KM268898, MF782478 |
| C | 6 | MH053308, KM268897, MH053309, AJ133357, MH053310, AJ007347 |
| O | 21 | AY593819, MH053313, MH053311, MH053312, KF112885, KJ206909, HQ632769, HQ632771, KU291242, KR401154, GU384683, KF694737, AJ539140, MH053315, JX040491, MH053317, MH053318, MH053316, KJ560291, DQ404170, KU821591 |
| SAT 1 | 19 | AY593838, AY593845, MH053319, AY593844, JF749860, MH053321, AY593846, AY593839, AY593842, AY593841, AY593840, MH053322, AY593843, KM268899, MH053323, MH053324, MH053325, MH053326, MH053327, MW355668, MW355669 |
| SAT2 | 15 | MH053330, MH053332, MH053328, MH053329, JX014255, MH053333, AY593849, JX014256, AY593847, MH053335, KM268900, JF749862, MH053336, MH053337, KU821592, MW355670 - MW355673 |
| SAT3 | 13 | AY593853, AY593851, MH053339, MH053340, MH053344, MH053343, AY593850, KJ820999, MH053341, MH053351, KX375417, KM268901, MH053350, MW355674 - MW355680 |
