# Supplementary material for: The RNA pseudoknots in foot-and-mouth disease virus are dispensable for genome replication, but essential for the production of infectious virus
Source: PLoS Pathog. 2022 Jun 6;18(6):e1010589. doi: 10.1371/journal.ppat.1010589 (PMC9203018; doi:10.1371/journal.ppat.1010589)
Supplement: S2 Table — (DOCX) [file ppat.1010589.s002.docx]

**S2 Table**

| **Nucleotide position downstream poly-C tract** | **Nucleotide** | **Experiments** | | | | **Average** |
| --- | --- | --- | --- | --- | --- | --- |
|  |  | **1** | **2** | **3** | **4** |  |
| 1 | A | 0 |  | 1.07 | 0.71 | 0.593333 |
| 2 | A | 0.6 |  | 1.46 | 0.65 | 0.903333 |
| 3 | G | 0 |  | 1.7 | 0.89 | 0.863333 |
| 4 | U | 0.13 |  | 0.53 | 0.3 | 0.32 |
| 5 | U | 0 |  | 1.05 | 0.66 | 0.57 |
| 6 | U | 0.36 |  | 0 | 0.05 | 0.136667 |
| 7 | U | 0.03 |  | 0.02 | 0.54 | 0.196667 |
| 8 | A | 0.35 |  | 0.25 | 0 | 0.2 |
| 9 | C | 0.47 | 1.04 | 1.04 | 0.59 | 0.785 |
| 10 | C | 0.83 | 1.03 | 1.07 | 1.13 | 1.015 |
| 11 | G | 0 | 0.08 | 0 | 0.15 | 0.0575 |
| 12 | U | 0 | 0 | 0 | 0 | 0 |
| 13 | C | 0 | 0 | 0 | 0 | 0 |
| 14 | G | 0.35 | 0.49 | 0.21 | 0.3 | 0.3375 |
| 15 | U | 1.63 | 2.52 | 2.26 | 2.28 | 2.1725 |
| 16 | U | 0.89 | 1.25 | 1.01 | 1.03 | 1.045 |
| 17 | C | 0.5 | 0.66 | 0.37 | 0.55 | 0.52 |
| 18 | C | 0.16 | 0.21 | 0.15 | 0.16 | 0.17 |
| 19 | C | 0.13 | 0.3 | 0.21 | 0.2 | 0.21 |
| 20 | G | 0 | 0 | 0 | 0 | 0 |
| 21 | A | 0.03 | 0 | 0 | 0.02 | 0.0125 |
| 22 | C | 0.08 | 0 | 0 | 0 | 0.02 |
| 23 | G | 0.51 | 0.37 | 0.16 | 0.43 | 0.3675 |
| 24 | U | 0.86 | 1.02 | 0.64 | 0.75 | 0.8175 |
| 25 | A | 0.45 | 0.95 | 0.62 | 0.64 | 0.665 |
| 26 | A | 0.16 | 0.38 | 0.46 | 0.21 | 0.3025 |
| 27 | A | 1.95 | 0 | 0.21 | 2.6 | 1.19 |
| 28 | A | 0.07 | 0.25 | 0.21 | 1.03 | 0.39 |
| 29 | G | 0.72 | 0 | 0 | 0.06 | 0.195 |
| 30 | G | 0 | 0 | 0.02 | 0.03 | 0.0125 |
| 31 | G | 0 | 0 | 0 | 0 | 0 |
| 32 | A | 0 | 0 | 1.13 | 0.97 | 0.525 |
| 33 | G | 0.73 | 0 | 0 | 0 | 0.1825 |
| 34 | G | 0.11 | 0.07 | 0.06 | 0.05 | 0.0725 |
| 35 | U | 0.55 | 0.93 | 0.98 | 0.56 | 0.755 |
| 36 | A | 0.68 | 1.11 | 1 | 0.77 | 0.89 |
| 37 | A | 0.45 | 0.6 | 0.81 | 0.55 | 0.6025 |
| 38 | C | 0.16 | 0 | 0.19 | 0.32 | 0.1675 |
| 39 | C | 0.15 | 0.34 | 0.5 | 0.39 | 0.345 |
| 40 | A | 0.01 | 0.07 | 0.08 | 0.02 | 0.045 |
| 41 | C | 0.41 | 0.33 | 0.46 | 0.43 | 0.4075 |
| 42 | A | 0.99 | 0.89 | 0.63 | 0.97 | 0.87 |
| 43 | A | 0.81 | 0.67 | 0.39 | 0.69 | 0.64 |
| 44 | G | 1.07 | 0.86 | 0.52 | 0.95 | 0.85 |
| 45 | C | 0 | 0 | 0 | 0 | 0 |
| 46 | U | 0.02 | 0.03 | 0.05 | 0.16 | 0.065 |
| 47 | U | 0.13 | 0.15 | 0 | 0 | 0.07 |
| 48 | G | 0.23 | 0.5 | 0.39 | 0.19 | 0.3275 |
| 49 | A | 0.6 | 1.18 | 0.91 | 0.94 | 0.9075 |
| 50 | A | 0.45 | 0.78 | 0.85 | 0.74 | 0.705 |
| 51 | A | 0.53 | 0.59 | 0.43 | 0.59 | 0.535 |
| 52 | C | 0.25 | 0.26 | 0 | 0.22 | 0.1825 |
| 53 | C | 0 | 0 | 0 | 0 | 0 |
| 54 | G | 0 | 0 | 0 | 0 | 0 |
| 55 | U | 0 | 0 | 0 | 0 | 0 |
| 56 | C | 0 | 0 | 0 | 0 | 0 |
| 57 | C | 0.46 | 0 | 0 | 0 | 0.115 |
| 58 | G | 0.35 | 0 | 0.31 | 0.42 | 0.27 |
| 59 | G | 0.24 | 0 | 0.17 | 0.26 | 0.1675 |
| 60 | C | 0.11 | 0 | 0.41 | 0.15 | 0.1675 |
| 61 | C | 0.03 | 0 | 0 | 0.01 | 0.01 |
| 62 | C | 0.03 | 0.02 | 0.02 | 0.02 | 0.0225 |
| 63 | G | 0.08 | 0.14 | 0.11 | 0.11 | 0.11 |
| 64 | A | 0.26 | 0.23 | 0.25 | 0.28 | 0.255 |
| 65 | C | 0.12 | 0.1 | 0.03 | 0.02 | 0.0675 |
| 66 | G | 0.54 | 0.71 | 0.5 | 0.53 | 0.57 |
| 67 | U | 0.11 | 1.23 | 0.92 | 1.09 | 0.8375 |
| 68 | A | 1 | 1.29 | 1.45 | 1.43 | 1.2925 |
| 69 | A | 0.84 | 0.13 | 0.17 | 0.18 | 0.33 |
| 70 | A | 0.03 | 0 | 0.7 | 0.19 | 0.23 |
| 71 | A | 1.05 | 0.9 | 1.08 | 1.5 | 1.1325 |
| 72 | G | 0.98 | 1.31 | 0.35 | 1.49 | 1.0325 |
| 73 | G | 0.17 | 0.36 | 0.11 | 0.02 | 0.165 |
| 74 | G | 0.01 | 0 | 0.05 | 0.1 | 0.04 |
| 75 | U | 1.81 | 1.53 | 1.64 | 2.21 | 1.7975 |
| 76 | G | 0.05 | 0 | 0.02 | 0 | 0.0175 |
| 77 | G | 0.06 | 0.11 | 0.04 | 0 | 0.0525 |
| 78 | U | 0.41 | 0.22 | 0.19 | 0.35 | 0.2925 |
| 79 | A | 0.51 | 0.51 | 0.49 | 0.56 | 0.5175 |
| 80 | A | 0.37 | 0.33 | 0.32 | 0.4 | 0.355 |
| 81 | C | 0.14 | 0.06 | 0.09 | 0.2 | 0.1225 |
| 82 | C | 0.19 | 0.3 | 0.44 | 0.29 | 0.305 |
| 83 | A | 0 | 0.03 | 0.17 | 0 | 0.05 |
| 84 | C | 0.13 | 0.06 | 0.19 | 0.03 | 0.1025 |
| 85 | A | 0.4 | 0.57 | 0.48 | 0.36 | 0.4525 |
| 86 | A | 0.3 | 0.53 | 0.38 | 0.36 | 0.3925 |
| 87 | G | 0.12 | 0.02 | 0 | 0 | 0.035 |
| 88 | C | 0 | 0.02 | 0.1 | 0.04 | 0.04 |
| 89 | U | 0.42 | 0.19 | 0.14 | 0.36 | 0.2775 |
| 90 | U | 1.13 | 0.82 | 0.52 | 1.12 | 0.8975 |
| 91 | A | 1.01 | 1.09 | 0.71 | 0.93 | 0.935 |
| 92 | C | 0.43 | 0.43 | 0.33 | 0.28 | 0.3675 |
| 93 | U | 0.5 | 0.8 | 0.74 | 0.48 | 0.63 |
| 94 | G | 0.44 | 0.57 | 0.5 | 0.45 | 0.49 |
| 95 | C | 0.19 | 0.16 | 0.08 | 0.12 | 0.1375 |
| 96 | C | 0 | 0 | 0 | 0 | 0 |
| 97 | G | 0.03 | 0.11 | 0 | 0 | 0.035 |
| 98 | U | 0.07 | 0.03 | 0 | 0 | 0.025 |
| 99 | C | 0.03 | 0 | 0.02 | 0 | 0.0125 |
| 100 | U | 0.14 | 0.24 | 0.21 | 0.22 | 0.2025 |
| 101 | U | 0.37 | 0.45 | 0.55 | 0.52 | 0.4725 |
| 102 | U | 0.11 | 0.19 | 0.15 | 0.11 | 0.14 |
| 103 | C | 0.03 | 0.02 | 0 | 0 | 0.0125 |
| 104 | C | 0.01 | 0 | 0 | 0 | 0.0025 |
| 105 | C | 0 | 0.02 | 0 | 0 | 0.005 |
| 106 | G | 0 | 0.02 | 0 | 0 | 0.005 |
| 107 | A | 0.24 | 0.11 | 0.07 | 0.07 | 0.1225 |
| 108 | C | 0.02 | 0 | 0 | 0 | 0.005 |
| 109 | G | 0.38 | 0.54 | 0.23 | 0.19 | 0.335 |
| 110 | U | 3 | 3 | 3 | 3 | 3 |
| 111 | U | 0.24 | 0.3 | 0.31 | 0.21 | 0.265 |
| 112 | A | 0.13 | 0.25 | 0.21 | 0.14 | 0.1825 |
| 113 | A | 0.18 | 0.39 | 0.43 | 0.31 | 0.3275 |
| 114 | A | 0.27 | 0.62 | 0.7 | 0.55 | 0.535 |
| 115 | G | 0 | 0 | 0 | 0 | 0 |
| 116 | G | 0 | 0 | 0 | 0 | 0 |
| 117 | G | 0 | 0 | 0 | 0 | 0 |
| 118 | A | 0.39 | 0 | 0 | 0 | 0.0975 |
| 119 | U | 1.56 | 0 | 0.8 | 0.57 | 0.7325 |
| 120 | G | 1.2 | 0.96 | 0.73 | 0.86 | 0.9375 |
| 121 | A | 1.23 | 0.81 | 0.81 | 0.92 | 0.9425 |
| 122 | A | 1.19 | 0.55 | 0.8 | 0.89 | 0.8575 |
| 123 | A | 0.57 | 0.12 | 0.36 | 0.46 | 0.3775 |
| 124 | C | 0.23 | 0 | 0.44 | 0.04 | 0.1775 |
| 125 | C | 0.38 | 0 | 2.15 | 0.38 | 0.7275 |
| 126 | A | 0.47 | 0.34 | 0.43 | 0.5 | 0.435 |
| 127 | C | 0.39 | 0.04 | 0.12 | 0.01 | 0.14 |
| 128 | A | 0.71 | 0.72 | 0.72 | 0.82 | 0.7425 |
| 129 | A | 0.88 | 0.81 | 0.72 | 0.93 | 0.835 |
| 130 | G | 1.09 | 0.97 | 0.73 | 0.99 | 0.945 |
| 131 | A | 0.67 | 0 | 0 | 0 | 0.1675 |
| 132 | C | 0.1 | 0 | 0.15 | 0.12 | 0.0925 |
| 133 | U | 0.02 | 0.02 | 0.06 | 0.03 | 0.0325 |
| 134 | U | 1.42 | 1.19 | 1.23 | 1.67 | 1.3775 |
| 135 | A | 2.13 | 1.94 | 2.22 | 2.76 | 2.2625 |
| 136 | C | 0 | 0 | 0 | 0 | 0 |
| 137 | C | 0 | 0 | 0.01 | 0 | 0.0025 |
| 138 | U | 0 | 0.11 | 0.06 | 0.07 | 0.06 |
| 139 | U | 0.29 | 0.42 | 0.3 | 0.29 | 0.325 |
| 140 | C | 0.31 | 0.33 | 0.26 | 0.31 | 0.3025 |
| 141 | G | 0 | 0 | 0.01 | 0 | 0.0025 |
| 142 | C | 0.19 | 0.18 | 0.14 | 0.14 | 0.1625 |
| 143 | U | 0.07 | 0.12 | 0.07 | 0.01 | 0.0675 |
| 144 | C | 0.06 | 0 | 0 | 0 | 0.015 |
| 145 | G | 0.22 | 0.08 | 0.23 | 0 | 0.1325 |
| 146 | G | 0.51 | 0 | 0 | 0 | 0.1275 |
| 147 | A | 0.65 | 0 | 0 | 0 | 0.1625 |
| 148 | A | 1.01 | 0 | 0 | 0 | 0.2525 |
| 149 | G | 1.38 | 0 | 0 | 0 | 0.345 |
| 150 | U | 1.39 | 0 | 0 | 0 | 0.3475 |
| 151 | A | 0.38 | 0.28 | 0.25 | 0.42 | 0.3325 |
| 152 | A | 0.21 | 0.11 | 0.25 | 0.3 | 0.2175 |
| 153 | A | 0.22 | 0 | 0 | 0 | 0.055 |
| 154 | A | 0.16 | 0 | 0.19 | 0.01 | 0.09 |
| 155 | C | 0.08 | 0 | 0 | 0 | 0.02 |
| 156 | G | 0.26 | 0 | 1.06 | 0.59 | 0.4775 |
| 157 | A | 0.57 | 0 | 0.44 | 0.24 | 0.3125 |
| 158 | C | 0.31 | 0.16 | 0.22 | 0.24 | 0.2325 |
| 159 | A | 0.94 | 0.48 | 0.8 | 0.71 | 0.7325 |
| 160 | A | 0 | 0.03 | 0.03 | 0.03 | 0.0225 |
| 161 | A | 0.1 | 0.14 | 0.33 | 0.28 | 0.2125 |
| 162 | C | 0 | 0.04 | 0.05 | 0 | 0.0225 |
| 163 | A | 0.06 | 0.21 | 0.33 | 0.17 | 0.1925 |
| 164 | C | 0.12 | 0.31 | 0.56 | 0.41 | 0.35 |
| 165 | A | 0.01 | 0.24 | 0.34 | 0.21 | 0.2 |
| 166 | C | 0.05 | 0.13 | 0.13 | 0.02 | 0.0825 |
| 167 | A | 0.9 | 1.07 | 1.21 | 1.2 | 1.095 |
| 168 | C | 0.61 | 0.46 | 0.49 | 0.37 | 0.4825 |
| 169 | A | 1.72 | 1.62 | 1.4 | 1.72 | 1.615 |
| 170 | G | 0.95 | 1.02 | 0.75 | 0.74 | 0.865 |
| 171 | U | 1.09 | 1.03 | 1.07 | 1.17 | 1.09 |
| 172 | U | 0.03 | 0.06 | 0.07 | 0.03 | 0.0475 |
| 173 | U | 0.09 | 0.04 | 0.04 | 0.05 | 0.055 |
| 174 | U | 0.1 | 0.06 | 0.06 | 0.06 | 0.07 |
| 175 | G | 0.03 | 0 | 0 | 0 | 0.0075 |
| 176 | C | 0.1 | 0 | 0 | 0 | 0.025 |
| 177 | C | 1.51 | 0.57 | 0.97 | 1.1 | 1.0375 |
| 178 | C | 0.09 | 0 | 0 | 0 | 0.0225 |
| 179 | G | 0.04 | 0 | 0 | 0 | 0.01 |
| 180 | U | 0 | 0 | 0.01 | 0 | 0.0025 |
| 181 | U | 0 | 0.02 | 0.01 | 0 | 0.0075 |
| 182 | U | 0.04 | 0.05 | 0.02 | 0.02 | 0.0325 |
| 183 | U | 0.17 | 0.14 | 0.19 | 0.04 | 0.135 |
| 184 | C | 0.65 | 0.48 | 0.38 | 0.27 | 0.445 |
| 185 | A | 1.54 | 1.46 | 1.48 | 1.33 | 1.4525 |
| 186 | U | 1.74 | 1.49 | 1.21 | 1.48 | 1.48 |
| 187 | G | 1.22 | 1.22 | 0.9 | 0.85 | 1.0475 |
| 188 | A | 0.52 | 0.46 | 0.16 | 0 | 0.285 |
| 189 | G | 1.2 | 1.33 | 1.22 | 0.15 | 0.975 |
| 190 | A | 0.23 | 0.06 | 0 | 1.18 | 0.3675 |
| 191 | A | 0.37 | 0.13 | 0.13 | 0.15 | 0.195 |
| 192 | A | 0.25 | 0 | 0.22 | 0.27 | 0.185 |
| 193 | U | 0.25 | 0.1 | 0.11 | 0.13 | 0.1475 |
| 194 | G | 0.04 | 0.06 | 0.12 | 0.14 | 0.09 |
| 195 | G | 0.22 | 0 | 0.02 | 0.01 | 0.0625 |
| 196 | G | 0 | 0 | 0.1 | 0 | 0.025 |
| 197 | A | 0 | 0 | 0 | 0 | 0 |
| 198 | C | 0.01 | 0 | 0.01 | 0 | 0.005 |
| 199 | G | 0.18 | 0 | 0 | 0 | 0.045 |
| 200 | U | 0.02 | 0 | 0 | 0 | 0.005 |
| 201 | C | 0.03 | 0.03 | 0.1 | 0 | 0.04 |
| 202 | U | 0.36 | 0.92 | 0.17 | 0.12 | 0.3925 |
| 203 | G | 1.09 | 0 | 1.03 | 0.9 | 0.755 |
| 204 | C | 0.03 | 0 | 0 | 0 | 0.0075 |
| 205 | G | 0 | 0.04 | 0 | 0 | 0.01 |
| 206 | C | 0.02 | 0.12 | 0.05 | 0 | 0.0475 |
| 207 | A | 0.15 | 0.34 | 0.01 | 0 | 0.125 |
| 208 | C | 0.45 | 0.23 | 0.03 | 0.07 | 0.195 |
| 209 | G | 0.44 | 0.02 | 0.27 | 0.2 | 0.2325 |
| 210 | A | 3 | 3 | 3 | 3 | 3 |
| 211 | A | 0.34 | 0.2 | 0.03 | 0.27 | 0.21 |
| 212 | A | 0.16 | 0.01 | 0.29 | 0 | 0.115 |
| 213 | C | 0 | 0 | 0.06 | 0 | 0.015 |
| 214 | G | 0.07 | 0 | 0.04 | 0.03 | 0.035 |
| 215 | C | 0 | 0 | 0 | 0 | 0 |
| 216 | G | 0 | 0 | 0 | 0 | 0 |
| 217 | C | 0 | 0 | 0 | 0 | 0 |
| 218 | C | 0.01 | 0.55 | 0.73 | 0.61 | 0.475 |
| 219 | G | 0.56 | 0.06 | 0 | 0.06 | 0.17 |
| 220 | U | 0.09 | 0 | 0.04 | 0 | 0.0325 |
| 221 | C | 0.26 | 0.11 | 0.08 | 0.17 | 0.155 |
| 222 | G | 0.6 | 0.06 | 0.15 | 0.21 | 0.255 |
| 223 | C | 0 | 0 | 0.11 | 0.07 | 0.045 |
| 224 | U | 0.08 | 0.03 | 0.14 | 0.07 | 0.08 |
| 225 | U | 0.09 | 0.03 | 0.28 | 0.49 | 0.2225 |
| 226 | G | 0.72 | 0.27 | 0.27 | 0.34 | 0.4 |
| 227 | A | 0.28 | 0.24 | 0 | 0 | 0.13 |
| 228 | G | 0.37 | 0 | 0.35 | 0.39 | 0.2775 |
| 229 | G | 0.04 | 0.3 | 0.02 | 0 | 0.09 |
| 230 | A | 0.06 | 0.01 | 0 | 0 | 0.0175 |
| 231 | G | 0.07 | 0.03 | 0.36 | 0.19 | 0.1625 |
| 232 | G | 0.64 | 0.01 | 0 | 0 | 0.1625 |
| 233 | A | 0.44 | 0.17 | 0 | 0 | 0.1525 |
| 234 | C | 0.19 | 0 | 0 | 0 | 0.0475 |
| 235 | U | 0 | 0 | 0 | 0 | 0 |
| 236 | U | 0 | 0 | 0 | 0 | 0 |
| 237 | G | 0 | 0 | 0 |  | 0 |
| 238 | U | 0 | 0 | 0.68 |  | 0.226667 |
| 239 | A | 0.14 | 0 |  |  | 0.07 |
