# Supplementary material for: The RNA pseudoknots in foot-and-mouth disease virus are dispensable for genome replication, but essential for the production of infectious virus
Source: PLoS Pathog. 2022 Jun 6;18(6):e1010589. doi: 10.1371/journal.ppat.1010589 (PMC9203018; doi:10.1371/journal.ppat.1010589)
Supplement: S3 Table — * P < 0.05, ** P < 0.01, *** P < 0.001 **** P < 0.0001, ns = not significant. (DOCX) [file ppat.1010589.s003.docx]

**S3 Table**

| **P0** | WT | Δ34 | Δ234 | C11 Δ1234 |
| --- | --- | --- | --- | --- |
| WT |  | ns | ns | * |
| Δ34 | ns |  | ns | *** |
| Δ234 | ns | ns |  | ** |
| C11 Δ1234 | * | *** | ** |  |
|  |  |  |  |  |
|  |  |  |  |  |
|  |  |  |  |  |
| **P1** | WT | Δ34 | Δ234 | C11 Δ1234 |
| WT |  | * | ns | *** |
| Δ34 | * |  | ns | *** |
| Δ234 | ns | ns |  | ** |
| C11 Δ1234 | *** | *** | ** |  |
|  |  |  |  |  |
|  |  |  |  |  |
| **P2** | WT | Δ34 | Δ234 | C11 Δ1234 |
| WT |  | ns | * | ** |
| Δ34 | ns |  | * | ** |
| Δ234 | * | * |  | ** |
| C11 Δ1234 | ** | ** | ** |  |
|  |  |  |  |  |
|  |  |  |  |  |
| **P3** | WT | Δ34 | Δ234 | C11 Δ1234 |
| WT |  | ns | * | ** |
| Δ34 | ns |  | * | ** |
| Δ234 | * | * |  | * |
| C11 Δ1234 | ** | ** | * |  |
