# Supplementary figures and images for: The RNA pseudoknots in foot-and-mouth disease virus are dispensable for genome replication, but essential for the production of infectious virus
Source: PLoS Pathog. 2022 Jun 6;18(6):e1010589. doi: 10.1371/journal.ppat.1010589 (PMC9203018; doi:10.1371/journal.ppat.1010589)

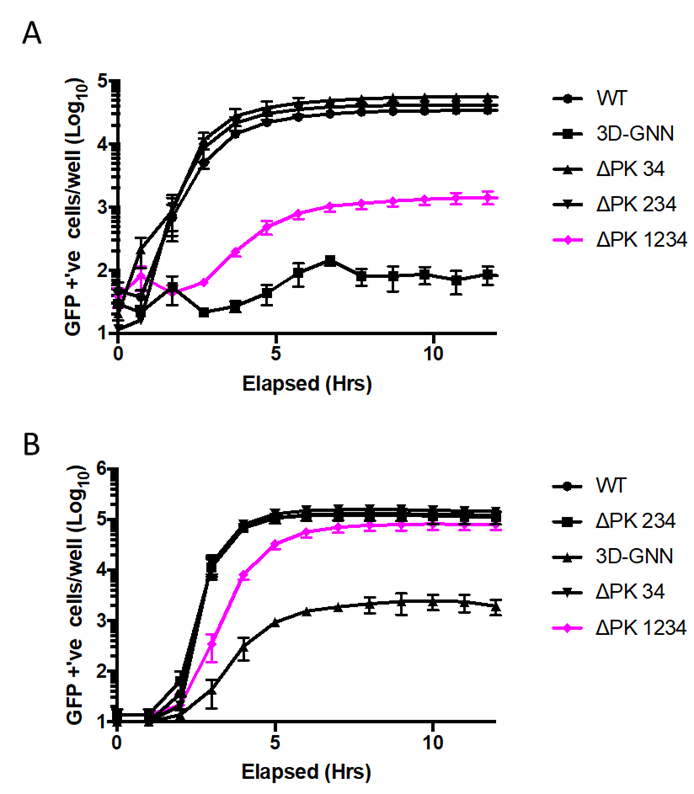

Supplement: S1 Fig — Replication of WT, 3D-GNN, ΔPK 34, ΔPK 234 and C11 ΔPK 1234 in MDBK and BHK-21 cells (A and B, respectively). GFP expression was monitored hourly for 12 hours using an IncuCyte Zoom. (n = 3), error bars represent SEM. (TIF) [file ppat.1010589.s004.tif]

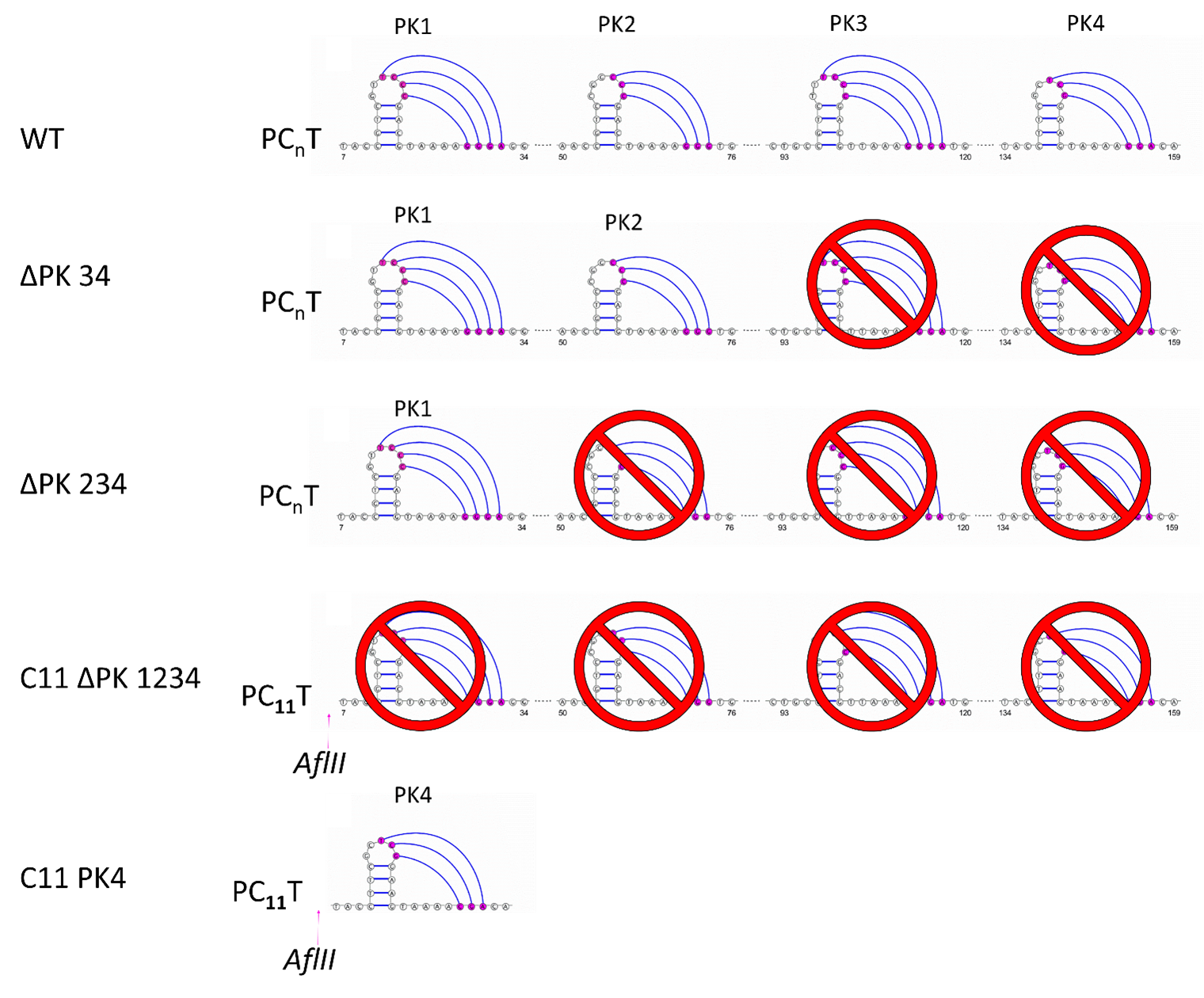

Supplement: S2 Fig — The length of poly-C-tract is represented as PCnT for wt and PC11T for truncated replicons. (TIF) [file ppat.1010589.s005.tif]

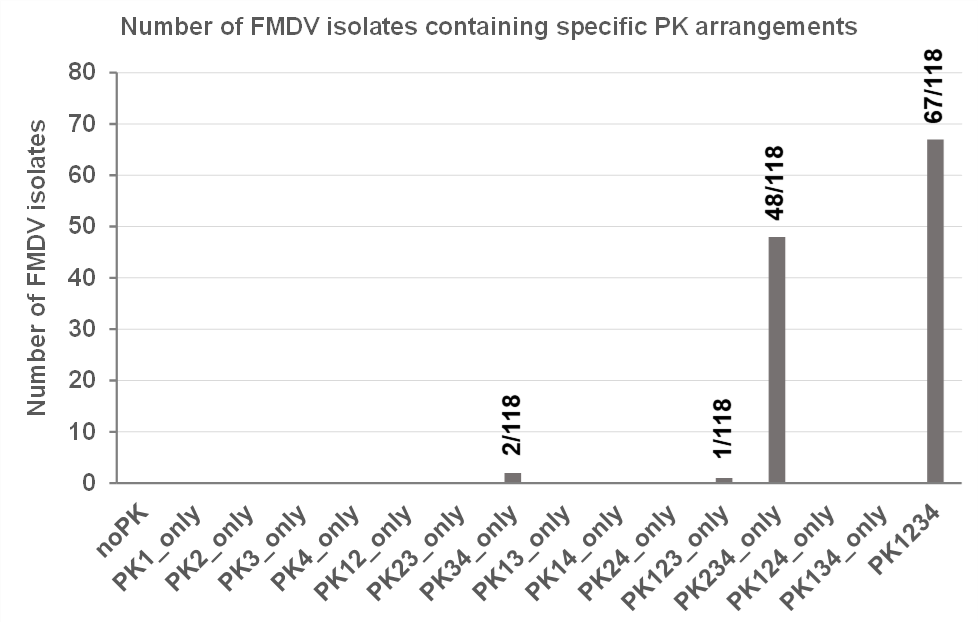

Supplement: S3 Fig — Genomic regions containing PKs of 118 FMDV isolates representing all 7 serotypes were aligned as described for Fig 2. The number of isolates containing specific PK arrangements (no PK; only a single PK present: PK1, PK2, PK3 or PK4; only two PKs present: PK12, PK23, PK34, PK13, PK14, PK24; only three PKs present: PK123, PK234, PK124, PK134; or all PKs present PK1234) was determined and visualised as number of isolates for each possible PK arrangement. Due to difficulty of sequencing through the poly-C tract, it is likely, at least for some of the isolates, that the lack of PK1 (which lies directly downstream of the poly-C tract) is a result of sequencing error. (TIF) [file ppat.1010589.s006.tif]
